# Sbp2l contributes to oligodendrocyte maturation through translational control in Tcf7l2 signaling

**DOI:** 10.1016/j.isci.2023.108451

**Published:** 2023-11-16

**Authors:** Masato Yugami, Yoshika Hayakawa-Yano, Takahisa Ogasawara, Kazumasa Yokoyama, Takako Furukawa, Hiroe Hara, Kentaro Hashikami, Isamu Tsuji, Hirohide Takebayashi, Shinsuke Araki, Hideyuki Okano, Masato Yano

**Affiliations:** 1 Research, Takeda Pharmaceutical Company Limited, 26-1 Muraoka-higashi 2-chome, Fujisawa, Kanagawa 251-8555, Japan; 2 Division of Neurobiology and Anatomy, Graduate School of Medical and Dental Sciences, Niigata University, 1-757, Asahimachidori, Chuo-ku, Niigata, Niigata 951-8510, Japan; 3 Department of Physiology, Keio University School of Medicine, 35 Shinanomachi, Shinjuku-ku, Tokyo 160-8582, Japan

**Keywords:** Biological sciences, Biochemistry, Cell biology

## Abstract

Oligodendrocytes (OLs) are the myelin-forming cells in the CNS that support neurons through the insulating sheath of axons. This unique feature and developmental processes are achieved by extrinsic and intrinsic gene expression programs, where RNA-binding proteins can contribute to dynamic and fine-tuned post-transcriptional regulation. Here, we identified SECIS-binding protein 2-like (Sbp2l), which is specifically expressed in OLs by integrated transcriptomics. Histological analysis revealed that Sbp2l is a molecular marker of OL maturation. Sbp2l knockdown (KD) led to suppression of matured OL markers, but not a typical selenoprotein, Gpx4. Transcriptome analysis demonstrated that *Sbp2l* KD decreased cholesterol-biosynthesis-related genes regulated by Tcf7l2 transcription factor. Indeed, we confirmed the downregulation of Tcf7l2 protein without changing its mRNA in *Sbp2l* KD OPCs. Furthermore, *Sbp2l* KO mice showed the decrease of Tcf7l2 protein and deficiency of OL maturation. These results suggest that Sbp2l contributes to OL maturation by translational control of Tcf7l2.

## Highlights

• *In silico* transcriptomic screen identified an oligodendrocyte-specific RBP, Sbp2l
• Sbp2l contributes to the oligodendrocyte maturation through Tcf7l2 signaling
• Sbp2l controls *Tcf7l2* mRNA translation through SECIS-like element in 3′UTR

## Graphical abstract

**Figure:**
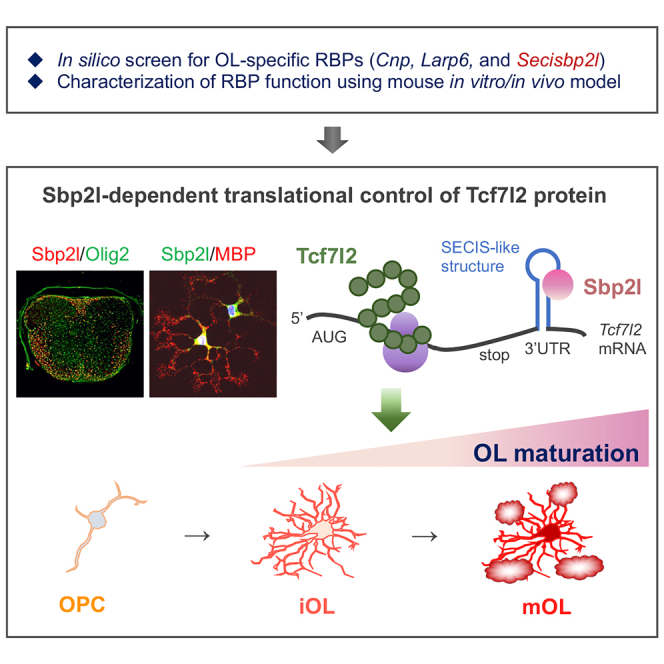

## Introduction

Oligodendrocytes (OLs) are glial cells in the central nervous system (CNS) that support neurons by forming myelin, which acts as an insulating sheath of axons. Several excellent studies using animal model systems, such as genetically modified animals and *in vivo* manipulation, and *in vitro* cultured oligodendrocyte precursor cells (OPCs) have helped to elucidate the underlying mechanisms of OL development from the patterning of the neuroepithelium and the specification of OPCs to the maturation of OLs: cooperative action of extrinsic signaling molecules and intrinsic transcriptional and epigenetic factors mediates the steps of this process.^1^^,^^2^^,^^3^^,^^4^^,^^5^ In the developing spinal cord, for example, the gradient of Sonic Hedgehog (SHH), a morphogen secreted from floor plate and notochord, gives rise to the pMN domain marked by the Olig2 transcription factor in the mouse and chick.^6^^,^^7^^,^^8^ The combinatorial action of transcription factors enables sequential production of motor neurons and then glial cells, including OLs and astrocytes.^9^ In addition, many secreted factors, such as growth factors, cytokines, and chemokines, and the downstream effectors contribute to OPC proliferation, OL differentiation, migration, and myelination *in vivo.*^2^^,^^10^

A recent study reported that approximately ∼1,542 genes encode RNA-binding proteins (RBPs), and their expression levels showed that the degree of tissue specificity of RBPs was lower than that of transcription factors.^11^ This evidence suggests that many RBPs support RNA processes that govern basic cellular events; however, cell-type specific RBPs also have pivotal roles in producing the cell-type-specific transcriptome and functional proteins in the development of CNS.^11^^,^^12^^,^^13^ In fact, neuron-specific Nova2 regulates the radial migration of postmitotic neurons, and Quaking5 (Qki5) protein contributes to the maintenance of neural stem cells.^14^^,^^15^ In addition, it is estimated that the correlation between global transcript and protein levels is ∼50% due to post-transcriptional regulation.^16^ In OLs, mRNAs of *Plp* and *Mbp* are already expressed without substantial translation, even in the late embryonic stage, but the protein expression was detected after birth. This finding suggests the importance of regulation at the post-transcriptional level in OLs. For example, Qki proteins are the best-characterized RBPs in OLs.^17^ Qki6/7 regulate mRNA stabilization and translation in the cytoplasm to precisely regulate the timing of differentiation.^18^ Several studies have used animals with spontaneous mutations to gain insight into the molecular mechanisms of hypomyelinating and demyelinating diseases.^19^^,^^20^ Indeed, dysfunction of OL development and demyelination can cause a wide variety of diseases, including developmental and adult-onset diseases, such as leukodystrophy, multiple sclerosis, and schizophrenia. More recently, OLs were suggested to play active roles in supporting neurons in terms of local energy metabolism by transporting lactate for the mitochondria in the axons.^21^ The function of myelinating OLs is linked to the condition and function of the myelinated axons of neurons. Therefore, understanding the precise mechanisms of OLs development and pathogenesis is essential for the development of new diagnostic and therapeutic strategies.

In the present study, we carried out a bioinformatic screening system adapted to the cell-type specificity index (pSI) method^22^ to identify new RBPs involved in OL development. As a result, we identified SECIS-binding protein 2 (Secisbp2l, in short Sbp2l) as a potential OL-specific RBP. The SECIS (Sec insertion sequence) element in 3′UTR selenoprotein mRNA enables to recode in-frame UGA codon from stop to selenocysteine. The RNA-binding protein Secisbp2 (Sbp2), which binds to this element, is essential for selenoprotein synthesis; Sbp2l was identified as its orthologous molecule.^23^ Detailed immunostaining using the developing mouse spinal cord revealed that Sbp2l is specific to OLs and colocalized with the OL marker protein, Qki7, also known as CC1.^24^ Loss-of-function analysis of Sbp2l and Sbp2 revealed that Sbp2l has a role in OL maturation distinct from that of Sbp2. Furthermore, transcriptome analysis with loss of Sbp2l function in OPCs suggested that Sbp2l contributes to the maturation of OLs and the cholesterol biosynthesis pathway. Consistent with this hypothesis, we confirmed that Sbp2l binds to the mRNA of *Tcf7l2*, a key transcription factor in cholesterol biosynthesis, and Sbp2l knockdown (KD) downregulates Tcf7l2 protein expression without changing Gpx4 protein levels in OPCs. In addition, Sbp2l systemic knockout (KO) mice exhibited a decrease in Tcf7l2 protein in OLs and abnormalities in OL maturation. Taken together, these data suggest that Sbp2l regulates OL-specific Tcf7l2 translation and contributes to cholesterol biosynthesis required for myelination and OL maturation.

## Results

### Identification of oligodendrocyte-specific RNA-binding proteins

**Figure 1:**
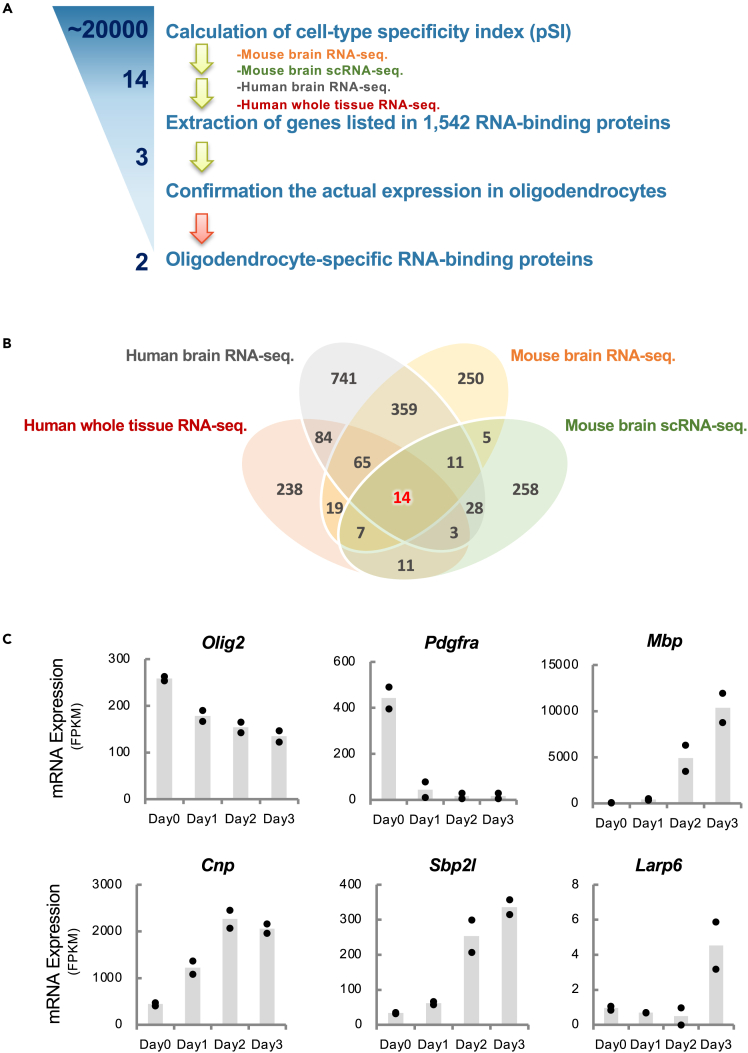
Identification of Sbp2l as an OL-specific RBP (A) Schematic illustration of the bioinformatic screening flow of OL-specific RBPs using the cell-type specificity index (pSI). (B) The Venn diagram displays overlaps of four different datasets and the numbers of OPC/OL-specific genes. See also Table S1. (C) Bar graphs showing the mRNA expression (FPKM) of OL lineage markers (*Olig2*, *Pdgfra*, *and Mbp*) and identification of RBPs (*Cnp*, *Sbp2l*, and *Larp6*) in OPC culture. Day represents the duration after the withdrawal of growth factors and the addition of T3. Note that transcript levels of *Sbp2l* are increased with OPC differentiation. Each dot represents two independent experiments.

**Figure 2:**
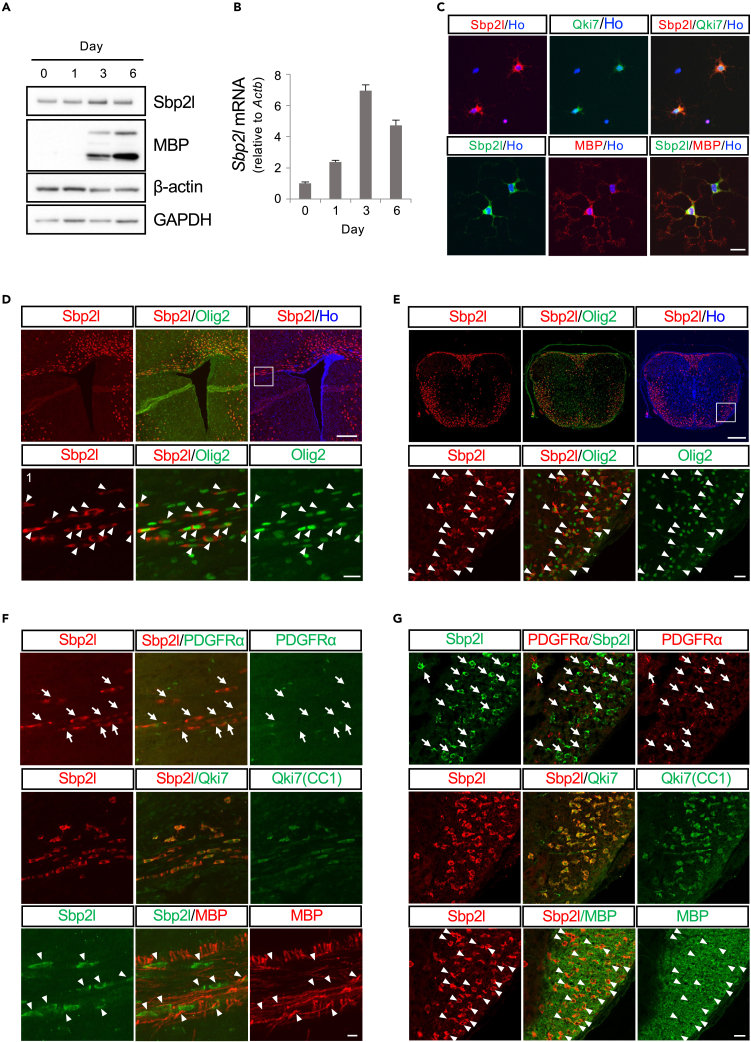
Sbp2l is selectively expressed in the OL cell linage (A) Western blot analysis of the protein expression of Sbp2l, MBP, β-actin, and GAPDH in OPCs *in vitro* with induction of OL differentiation. Day indicates the days after the induction of OL differentiation. See also Figure S1A. (B) *Sbp2l* mRNA expression relative to *Actb.* Data represent the mean ± SD of four replicates. (C) Immunocytochemistry using antibodies against Sbp2l protein (red) and Qki7 (CC1) (green) combined with Hoechst dye (blue) to detect nuclei in OPCs/OLs at day 3 after induction of OL differentiation (top). Similarly, Sbp2l protein (green) was observed in MBP-positive OLs (red) (bottom). Scale bar: 20 μm. See also Figure S1B. (D) Immunofluorescence staining using antibodies against Sbp2l (red) and OPC/OL lineage marker Olig2 (green). Sbp2l is expressed in Olig2-positive OL-lineage cells in the corpus callosum in P14 mouse brain (top). Hoechst dye (blue) is used for the detection of the nuclei. Enlarged view images of the boxed area are shown in the bottom panel. Double-positive cells indicated by arrowheads (bottom). Scale bars: 200 μm for low magnification and 20 μm for high magnification. (E) Similarly, Sbp2l-positive cells are mainly observed in the white matter of the spinal cord. Immunofluorescence staining was performed on mouse spinal cord transverse sections at P7 using antibodies against Sbp2l (red) and Olig2 protein (green) in the top panel. An enlarged view is shown in the bottom panel. Double-positive cells indicated by arrowheads. Scale bars: 200 μm for low magnification and 20 μm for high magnification. (F) Immunofluorescence staining using antibodies against Sbp2l and OPC/OL-lineage marker proteins in mouse brain at P14. Sbp2l expression (red) was well colocalized with Qki7 (CC1, green) in the middle. Sbp2l (green) was also found in MBP-positive OLs (red) indicated by arrowheads (bottom). However, Sbp2l (red)-positive cells are not expressing PDGFRα (green), as indicated by arrows (top panel). Scale bar: 20 μm. See also Figure S1C. (G) Immunofluorescence staining on mouse spinal cord sections at P7. Sbp2l (red) is well colocalized with Qki7 and MBP-positive OLs (green) (middle and bottom panels). Arrowheads represent Sbp2l/MBP-positive cells (bottom). Sbp2l (green) is not expressed in PDGFRα-positive OPCs (red), as indicated by arrows (top panel). Scale bar: 20 μm.

To identify OL-specific RBPs for both humans and mice, we first explored tissue- or cell-type-specific genes by calculating specificity indexes for 3 publicly available human and mouse brain datasets^25^^,^^26^^,^^27^ using the pSI (specificity index p values) package (Figure 1A).^22^ In addition, nervus system specificity over whole tissues was considered by using the reported pSI for whole human tissues using GTEx data.^28^ By merging significant genes in these 4 datasets (pSI <0.05), 14 genes were identified as OL-specific genes, which included genes encoding well-known OL lineage marker proteins, myelin basic protein (MBP), and Olig2 (Figure 1B; Table S1). Among them, only 3 RBPs, *Cnp*, *Sbp2l*, and *Larp6*, were found in the potential OL-specific RBPs by referring to the∼1,542 RBPs catalog.^11^ Among those, 2′,3′-cyclic nucleotide 3′-phosphodiesterase (CNPase, encoded by *Cnp* gene) that is expressed exclusively in myelin-forming OLs in the CNS is essential for myelin formation in mice and humans.^29^^,^^30^ This evidence reflects the validity of our bioinformatic prediction. To confirm their gene expression profiles in OL lineage cells, we performed time-course mRNA-sequence analyses obtained from mouse primary cultured OPCs, which were differentiated into OLs under the differentiation conditions (Figure 1C). The data recapitulated the OL differentiation such that *Pdgfra* and *Olig2* were downregulated and *Cnp* and *Mbp*, an OL gene, were upregulated during the differentiation. Interestingly, *Sbp2l* showed an increase of mRNA expression similar to OL genes, which implicated that Sbp2l also plays a role in myelin formation. Sbp2l was originally identified as a paralog of Sbp2 in vertebrates by the sequence similarity of the C-terminal 429 amino acid sequence of Sbp2 bearing 46% identity.^23^ Although its function has long been unknown, Sbp2l has only recently been reported to be highly expressed in OLs and associated with OL differentiation.^31^ Next, to investigate the detailed protein expression profile *in vitro* and *in vivo*, we generated anti-Sbp2l antibodies using a peptide and a recombinant protein for mouse Sbp2l as antigens. Both antibodies for Sbp2l specifically detected this protein in mouse wild-type (WT) primary OPC/OLs but not in *Sbp2l* KD OPC/OLs (Figure S1A). Both Sbp2l mRNA and protein expression were increased and peaked at day 3 in primary OPC/OLs (Figures 2A and 2B). Furthermore, immunofluorescence staining using anti-Sbp2l antibodies indicated that Sbp2l is expressed in Qki7 (CC1)-positive and MBP-positive OLs at 3 days after differentiation *in vitro*. Sbp2l is dominantly localized in the cytoplasm and the proximal region of OLs, whereas the mature OL marker, MBP, reflects the extent of maturation and the whole morphology of OLs (Figure 2C). We further confirmed that Sbp2l expression was observed in Olig2-positive OL-lineage cells and gradually upregulated from PDGFRα-positive OPC to PDGFRα-negative OLs in an *in vitro* culture system (Figure S1B).

To examine the detailed expression of Sbp2l protein *in vivo*, we performed immunofluorescence staining using the Sbp2l-specific antibodies in postnatal day 14 (P14) mouse brain and P7 mouse spinal cord tissues. Sbp2l appeared obviously in the corpus callosum (CC) of forebrain and the white matter of the thoracic spinal cord. Double staining showed that Sbp2l is expressed in the majority of Olig2-positive glial lineage cells in the white matter (Figures 2D and 2E). The expression pattern of Sbp2l protein is well colocalized with the OL-differentiating marker, Qki7. However, Sbp2l was not detectable in PDGFRα-positive OPCs and GFAP- and glutamine synthetase (GS)-positive astrocytes (Figures 2F, 2G, and S1C) *in vivo*. These data suggest that Sbp2l is expressed in mature OLs expressing MBP, a component of myelin. This finding is consistent with the results of primary OPCs *in vitro* in this study and Dai et al.^31^ and supports that Sbp2l is an RBP specifically expressed in differentiating OLs, as predicted by our bioinformatic screen, and could be a useful marker for differentiating OLs. These data strongly suggest that Sbp2l plays a role in OL maturation and myelination.

### Sbp2l functions in oligodendrocyte maturation

**Figure 3:**
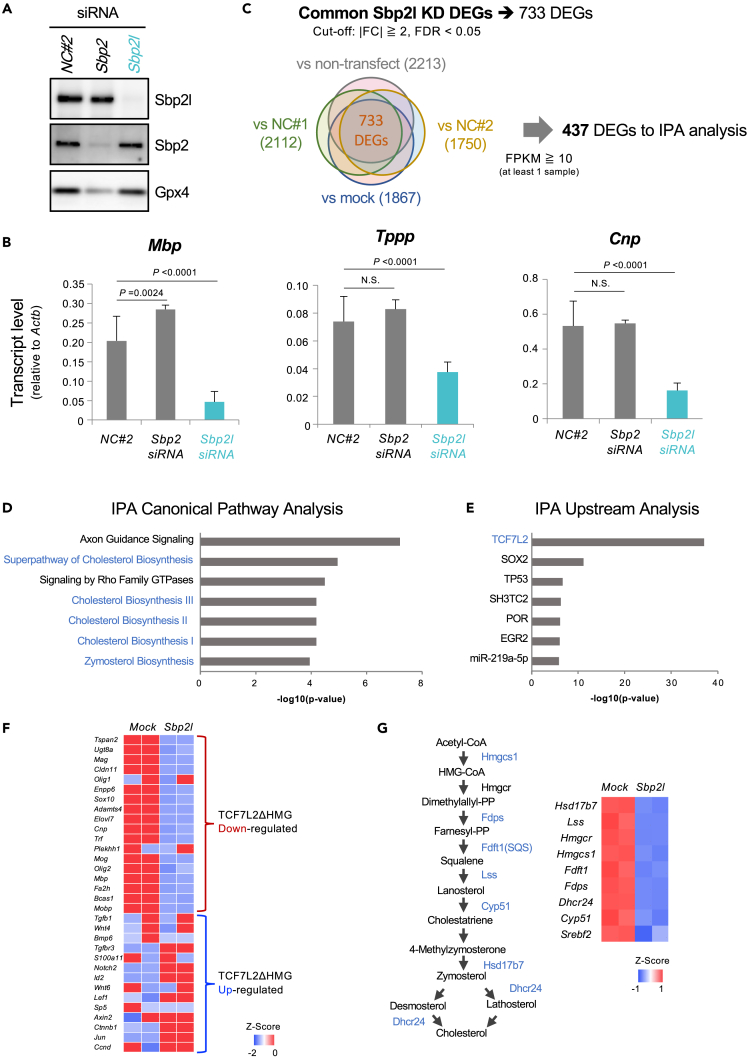
*Sbp2l* KD OPCs cause downregulation of genes associated with OL differentiation and cholesterol biosynthesis (A) Western blots confirmed the reduction in Sbp2l and Sbp2 protein in each KD OPC. Note that the Gpx4 protein level remained in *Sbp2l* KD cells but not in *Sbp2l* KD cells. (B) RT-qPCR indicates that *Sbp2l* KD, but not *Sbp2* KD, affects the expression of OL differentiation in OPC. Bar graphs showing the relative transcript level to *Actb.* Data represent the mean ± SD of three replicates. Adjusted p value by Dunnett’s test. (C) A Venn diagram showing the number of DEGs between *Sbp2l* KD and each control (cutoff: |FC| ≧ 2, FDR <0.05) and common DEGs between the KD and control groups. Then, only genes filtered by FPKM ≧ 10 (at least 1 sample) were considered common DEGs and used for subsequent pathway analyses. See also Table S1. (D) IPA canonical pathway analysis revealed that cholesterol-biosynthesis-pathway-related genes were significantly changed in the *Sbp2l* KD OPCs. Values represent -log10 (p value). Blue color indicates the cholesterol-synthesis-related pathway. See also Figures S2A and S2B. (E) IPA upstream analysis predicted that the TCF7L2 transcriptional factor was a top upstream gene for the DEGs. Values represent -log10 (p value). Blue color indicates cholesterol-synthesis-related genes. (F) The heatmap indicates transcript level changes in OPC-/OL-related genes in *Sbp2l* KD OPC compared with DEGs in *TCF7l2* ΔHMG cKO. (G) The heatmap indicates that transcript level changes in cholesterol-biosynthesis-related genes in *Sbp2l* KD OPCs (right). Downregulated genes in the cholesterol biosynthesis pathway (left). Blue represents decreased genes in *Sbp2l* KD.

Sbp2l is the paralog of Sbp2 and binds to SECIS-containing sequences.^23^ However, it lacks the ability to catalyze incorporation of selenocysteine (Sec) into the proteins.^32^^,^^33^ Next, to investigate Sbp2l functions in OLs, we conducted siRNA-mediated KD experiments for *Sbp2l* as well as *Sbp2* in cultured OPCs (Figure 3A). We confirmed that both Sbp2 and Sbp2l proteins were expressed in OLs and that their expression was diminished in each KD cell (Figure 3A). Gpx4, a well-known selenoprotein, was clearly reduced in *Sbp2* KD OLs, which indicated that Gpx4 was converted to the truncated form due to the lack of Sec insertion; however, this reduction was not observed in *Sbp2l* KD OLs (Figure 3A). In contrast, RT-qPCR analysis demonstrated that a reduction in OL maturation markers, such as *Mbp*, *Cnp*, and *Tppp*, was observed in *Sbp2l* KD but not in *Sbp2* KD (Figure 3B). These results suggest that Sbp2l does not share a function with Sbp2 and has a unique role in OL differentiation independent of its Sec incorporation function.

To decipher the mechanism of OL maturation by Sbp2l, we performed mRNA-seq profiling of OPC/OLs (Figure 3C). We identified 733 common differentially expressed genes (DEGs) between the *Sbp2l* KD and nontreated, mock, and non-targeting siRNA #1 and #2 groups, and 10 or over FPKM counts in at least 1 sample were used for filtering. Finally, we obtained 437 DEGs out of 23,427 genes as a Sbp2l impacting gene signature (360 downregulated and 77 upregulated genes in *Sbp2l* KD OPCs) (Table S2). The following IPA canonical pathway analysis demonstrated that the cholesterol biosynthesis pathway was recurrently enriched in the *Sbp2l* KD OPCs (Figures 3D, S2A, and S2B). Interestingly, accompanied IPA upstream analysis for the upstream regulators in the dysregulated RNA network strongly predicted that Tcf7l2 is the top significant regulator for some DEGs (Figure 3E). In fact, Tcf7l2 is a transcription factor harboring HMG (high-mobility group) DNA-binding domain. Analyses using OL lineage-specific Tcf7l2 mutant mice without the HMG domain (*Tcf7l2*^fl/fl^:*Olig1*-Cre^+/−^, shortly *Tcf7l2*ΔHMG) indicated that Tcf7l2 regulated OL maturation and cholesterol biosynthesis pathway.^34^ Given that Sbp2l contributes to the Tcf7l2 function, we further examine the overlapping DEGs among *Sbp2l* KD and *Tcf7l2*ΔHMG. We found the DEGs in *Sbp2l* KD OPCs, including OL and cholesterol-biosynthesis-related genes, significantly overlapped with those of *Tcf7l2 ΔHMG* cKO (Figures 3F and 3G).^34^

### Sbp2l function *in vivo* using systemic Sbp2l knockout mice

**Figure 4:**
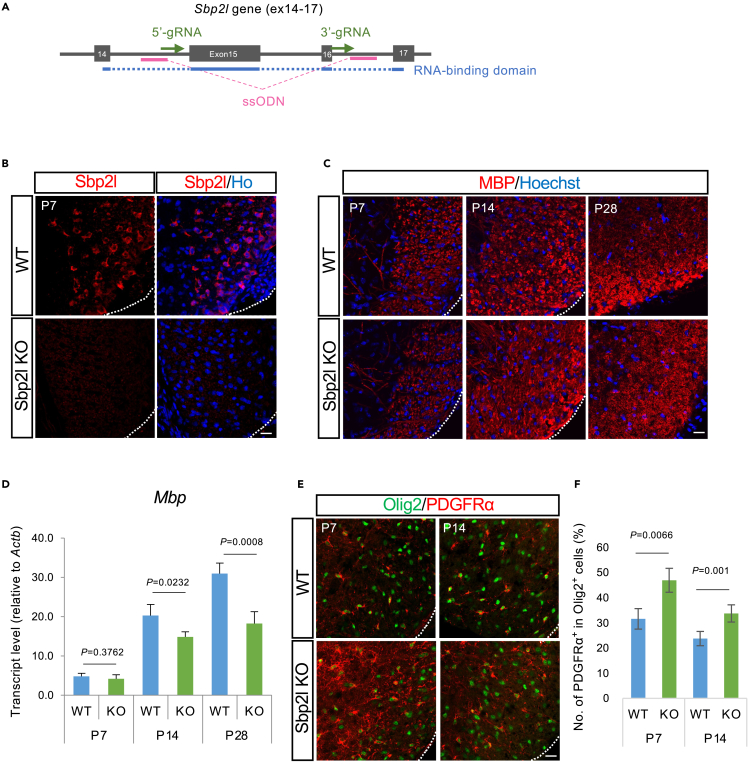
Sbp2l KO mice exhibited a defect in OL maturation (A) Schematic illustration of mouse genome editing using the CRISPR/Cas9 system for *Sbp2l* total KO mice. ssODN, single-stranded oligodeoxynucleotide. (B) Immunofluorescence staining was performed on mouse spinal cord transverse sections at P7 using antibodies against Sbp2l (red) with Hoechst dye (blue). Sbp2l protein was undetectable in the P7 *Sbp2l* mutant spinal cord. Scale bar indicates 20 μm. (C) Time course of MBP protein expression. Immunostaining for MBP (red) in the *Sbp2l* KO and WT spinal cord at the developing stage showing subtle changes in the MBP protein. Scale bar indicates 20 μm. See also Figures S3A–S3D. (D) RT-qPCR confirmed the significant downregulation of *Mbp* mRNA level in the spinal cord tissues of P14 and P28 *Sbp2l* null mice. Data represent the mean ± SD of four independent experiments. Welch’s t test*.* See also Figure S3E. (E) Immunofluorescence staining for Olig2 (green) combined with PDGFRα (red) in mouse spinal cord transverse sections at P7 and P14. Loss of the Sbp2l protein caused an increase in the ratio of PDGFRα/Olig2 double-positive OPCs in Olig2-positive cells at P7 and P14. Scale bar indicates 20 μm. (F) Bar graphs showing the ratio of PDGFRα-positive to Olig2-positive cells at P7 and P14 in (E). Quantitative analysis revealed a significant increase in PDGFRα/Olig2-double-positive OPCs in the *Sbp2l* mutant spinal cord. Data represent the mean ± SD of three independent experiments. Two-tailed Student’s t test*.*

To further explore the function of Sbp2l *in vivo*, we generated systemic KO mice using a CRISPR-Cas9 system, given the OL-specific expression (Figure 4A). Immunofluorescence analysis confirmed that the Sbp2l protein was completely lost at least by P7 in the KO spinal cord (Figure 4B). We performed immunohistochemistry using antibodies against PDGFRα and Olig2 as OPC marker proteins and MBP and PLP (proteolipid protein) as mature OL markers to search for any defects in OL-lineage cells at the developing stage in *Sbp2l* KO mice. Immunohistochemical analysis did not detect obvious changes in mature OL proteins, such as MBP and PLP, in the *Sbp2l* KO spinal cord (Figures 4C and S3A). This finding could be due to their subtle changes or structural protein properties. Thus, we performed Western blots as a semi-quantitative assay to detect changes in myelin protein and found that MBP protein was significantly decreased in *Sbp2l* KO (Figures S3B and S3C). FluoroMyelin assay, which can detect higher lipid content in myelin, also confirmed the significant decrease of myelin signal between WT and *Sbp2l* KO (Figure S3D). In addition, RT-qPCR analysis to observe the expression of mature OL genes showed that *Mbp* and *Mobp* (myelin-associated oligodendrocyte basic protein) mRNA expression was significantly downregulated in the P14 and P28 *Sbp2l* KO spinal cord (Figures 4D and S3E). On the other hand, the percentage of PDGFRα-positive OPCs in Olig2-positive cells was significantly increased in the P7 and P14 *Sbp2l* KO spinal cord compared with that of the control (Student’s t test, 1.48- and 1.42--fold, p = 0.0066 and p = 0.001, respectively) (Figures 4E and 4F). This phenotype, the increased number of OPCs, suggested the endogenous compensation for the maturation abnormality in OL. These data were largely compatible with the previous *Sbp2l* cKO analysis.^31^

### Sbp2l regulates the level of Tcf7l2 protein

**Figure 5:**
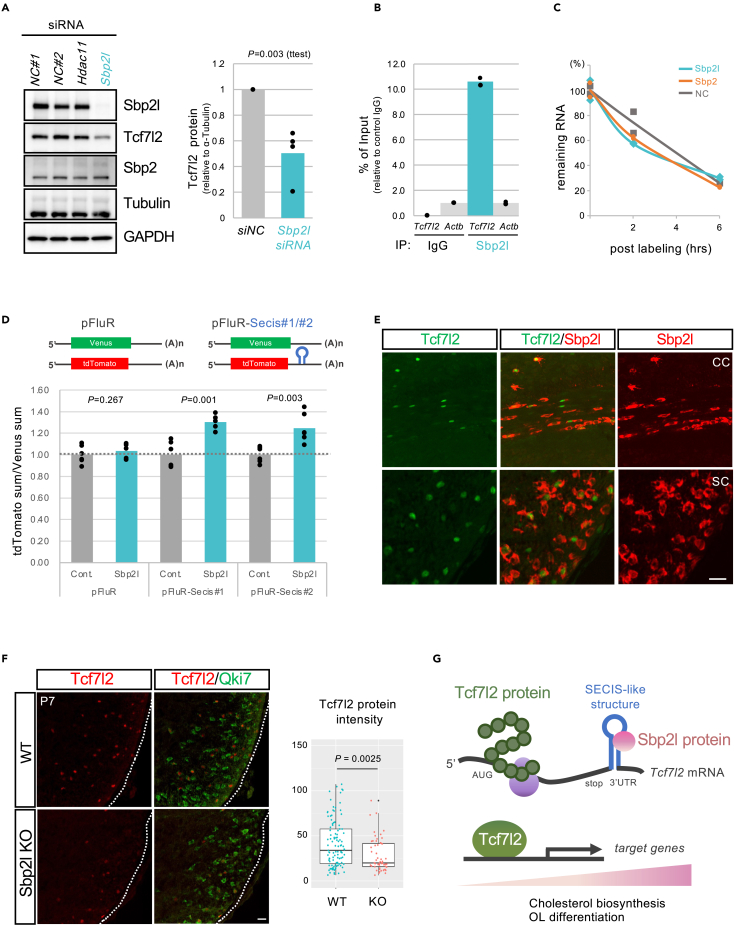
Sbp2l regulates Tcf7l2 protein levels by binding to the *Tcf7l2* 3′UTR (A) Western blots indicate that *Sbp2l* KD led to a reduction in Tcf7l2 protein expression in OPCs (left). Quantification of Tcf7l2 protein expression relative to α-Tubulin (right). Data represent mean of four independent experiments indicated by dots in the bar graph. Student’s t test. See also Figures S4A and S4B. (B) CLIP-qPCR assay using two different anti-Sbp2l antibodies indicating that Sbp2l efficiently binds to the *Tcf7l2* mRNA compared with that of control IgG. Data represent the percentage of input of *Tcf7l2* and *Actb* levels normalized to control IgG. Each dot represents an independent experiment. See also Figure S4C. (C) RNA labeling assay shows no difference in *Tcf7l2* mRNA stabilization between *Sbp2l* KD, *Sbp2l* KD, and control siRNA in OPCs. (D) Dual color reporter assay for SECIS-like sequence in 3′UTR of *Tcf7l2* mRNA. HeLa Tet-On 3G cells were transfected with pTRE-Tight-BI-Venus-tdTomato-Secis#1/#2 or pTRE-Tight-BI-Venus-tdTomato control vector together with Sbp2l expression or control vector and treated with doxycycline (10 ng/mL) to induce the expression. The relative signal intensity of the tdTomato to Venus fluorescent protein was calculated for each cell. Data represent mean of the TdTomato/Venus of three-independent experiments indicated by dots. Student’s t test. See also Figure S4D–F. (E) Tcf7l2 (green) is expressed in Sbp2l-positive OLs (red). Immunofluorescence was performed on the transverse sections of the brain at P14 (top) and the spinal cord at P7 (bottom). Scale bar: 20 μm. See also Figure S4G. (F) The Tcf7l2 protein expression (red) in Qki7-expressing OLs (green) on the transverse sections of the spinal cord at P7 (left). Decreased Tcf7l2 protein expression in the *Sbp2l* KO spinal cord compared with that of the control. Scale: 20 μm. Quantitative analysis of Tcf7l2 protein intensity in the spinal cord sections (right). Box and whisker plots showing Tcf7l2 protein intensity in the *Sbp2l* KO spinal cord compared with that of the control. Data represent the mean ± SD of four independent experiments. Each dot represents an immunofluorescence signal for the Tcf7l2 protein. Mann-Whitney U test*.* See also Figure S4H. (G) Model for OL-specific translational control of Tcf7l2 protein by Sbp2l in selenoprotein-independent manner. Ubiquitously expressed Tcf7l2 protein, a master regulator of cholesterol biosynthesis genes, is translationally controlled by Sbp2l through binding to the SESIS-like structure in the 3′UTR of *Tcf7l2* mRNA in OLs. Increased Tcf7l2 protein expression achieves the OL maturation and cholesterol biosynthesis required for subsequent myelination. See also Figure S5.

The above data indicate that Sbp2l is an RBP that is deeply involved in OL maturation *in vivo* and *in vitro* and that this function could be mediated by the Tcf7l2 protein according to bioinformatic prediction. Indeed, we found that the protein level of Tcf7l2 was significantly reduced in *Sbp2l* KD but not in *Sbp2* KD, although the *Tcf7l2* transcript was not changed in *Sbp2l* KD OPCs (Figures 5A or S4A and S4B). These results suggest that Sbp2l contributes to OL differentiation through Tcf7l2 protein expression in a selenoprotein-synthesis-independent manner. To investigate how Sbp2l controls the protein level of Tcf7l2, we performed a CLIP-qPCR assay to detect the direct interaction between Sbp2l and *Tcf7l2* mRNA *in vivo*.^35^ CLIP-qPCR assays using two distinct anti-Sbp2l antibodies revealed that *Tcf7l2* mRNA was efficiently associated with the Sbp2l protein (Figure 5B). Because Sbp2l is likely to bind the SESIS-like element, we used Rochester’s computational prediction^36^ to search for the SECIS-like structure in this mRNA, although Tcf7l2 was not included in a list of selenocysteine-containing proteins identified by previous studies.^37^^,^^38^ Interestingly, computational analysis predicted that the 3′-UTR of *Tcf7l2* mRNA would form a three-stem-containing hairpin structure similar to SECIS element (Figure S4C). We next performed a pulse-chase study using ethylene uridine (EU) to assess the involvement of Sbp2l in the half-life of *Tcf7l2* mRNA. However, we did not observe a difference in the mRNA decay rate between the control and *Sbp2l* KD cells (Figure 5C). Next to assess the action of Sbp2l on SECIS-like element-dependent translation, we used the cell-based color reporter system that we previously published.^39^ This Dox inducible birectional reporter in HeLa Tet-On 3G cells demonstrated that tdTomato expression wih a SECIS-like element of the 3'UTR of *Tcf7l2* mRNA was significantly increaased compared to control, suggesting that this SECIS-like element in *Tcf7l2* 3'-UTR promoted the translation (Figure 5D). In addition, we conducted cycloheximide (CHX) chase assay to assess the possibility of enhanced degradation of Tcf7l2 protein. Treatment of CHX, an inhibitor of eukaryotic protein synthesis, did not change the degradation rate of Tcf7l2 protein level between control and *Sbp2l* KD OPC cells (Figures S4D and S4E), indicating that Sbp2l did not stabilize Tcf7l2 protein. These results suggest a possible mechanism by which Sbp2l binds to *Tcf7l2* mRNA directly and controls Tcf7l2 protein synthesis at the translational level. We also found that forced expression of Tcf7l2 partially rescues the changes in mRNA expression of *Mobp*, *ccnd1*, *Jun*, and other mRNAs in *Sbp2l* KD OPCs (Figure S4F). Tcf7l2 is a transcriptional factor that is transiently expressed to regulate the oligodendrocyte maturation.^5^^,^^40^ We have also confirmed that Sbp2l protein is expressed in the Tcf7l2-positive cells of postnatal mouse brain, spinal cord, and cultured OPC/OLs (Figures 5E and S4G). We found that the protein level of Tcf7l2 was decreased in *Sbp2l* KO spinal cord without a change in *Tcf7l2* mRNA levels (Figures 5F and S4H). The intensity of the Tcf7l2 signal in the *Sbp2l* KO spinal cord was significantly decreased compared with that in the control spinal cord (Mann-Whitney U-test p = 0.0025) (Figure 5F), suggesting that Sbp2l is required for the synthesis of Tcf7l2 protein in OLs *in vivo* and proper OL maturation (Figure 5G). Taken together, these data suggest that Sbp2l regulates the expression of Tcf7l2 protein, a master regulator of cholesterol biosynthesis genes, in an OL-specific manner. Sbp2l could achieve the maturation and cholesterol biosynthesis required for subsequent myelination by OL-specific control of Tcf7l2 protein levels.

## Discussion

In the present study, we searched for RBPs specifically expressed in the OL cell lineage by analyzing various transcriptome datasets. We found a few RBPs, including *Cnp*, *Sbp2l*, and *Larp6*. Among them, we focused on Sbp2l because its protein expression is abundant and highly specific in the OL cell lineage, and this function has not been characterized at this point. Using a primary cultured OPC/OL model and postnatal mouse spinal cord, we showed that Sbp2l is an RBP whose expression increases dramatically during OL differentiation. Furthermore, mouse genetics with mRNA-seq analysis revealed an impact on OL maturation through molecules involved in the Tcf7l2-dependent cholesterol biosynthesis pathway. Finally, we found that Sbp2l contributes to the OL-specific translational control of the Tcf7l2 protein by binding to the SESIS-like structure in the 3′UTR of *Tcf7l2* mRNA. This regulation might to be independent of selenoprotein synthesis, because at least Tcf7l2 protein is not included in a list of selenoproteins identified by previous studies.^37^^,^^38^

We have focused on the cell-type specificity of RBPs and the role of their proteins in cellular functions and the regulatory transcriptome.^14^^,^^15^^,^^41^ These studies have enabled us to understand how RBPs contribute to the transcriptome and their physiological functions in specific cell types. Among them, Nova proteins and neuronal embryonic lethal abnormal visual proteins, which are identified as target antigens of paraneoplastic autoimmune diseases, are also neuron-specific molecules.^42^^,^^43^^,^^44^ RbFox3, which is one of the antigens of the representative neuronal marker antibody, NeuN, is also a neuronal RBP.^45^ In addition, Musashi1, PTBP1, and Qki5 have been studied as neural stem cell factors.^14^^,^^46^^,^^47^^,^^48^^,^^49^ Furthermore, the Qki protein is encoded by a gene responsible for the spontaneous mutant, *quaking viable*, and is involved in post-transcriptional regulation of its target genes that are widely important for OL differentiation.^17^^,^^18^ In general, they are involved in the generation of cell-specific transcriptomics through post-transcriptional gene regulation based on their specific expression.^50^ In the present study, we searched for RBPs focusing on the expression profile in OL differentiation to understand the OL-lineage-specific post-transcriptional program. We found a few subsets of RBPs, *Cnp*, *Sbp2l*, and *Larp6*. In particular, *Cnp*, encoding CNPase, is a classical OL-specific protein.^51^ CNPase is an enzyme that catalyzes the phosphodiester hydrolysis of 2′,3′-cyclic nucleotides to 2′-nucleotides and is also classified as an RBP, but its role in OL function and differentiation via nucleic acid metabolism remains obscure.^51^ Another top molecule in our screening is *Sbp2l*, a paralog of Sbp2.^52^ A very recent study revealed that Sbp2l is an OL-cell-lineage-specific molecule and regulates OL differentiation through the function of DIO-2 selenoprotein synthesis, leading to the production of thyroid hormone, T3.^21^^,^^31^ We have newly generated antibodies for Sbp2l to confirm its specific expression in the OL cell lineage. Sbp2l showed similar expression in the maturation process with the CC1 antibody, whose antigen is Qki7, which is important in the OL maturation.^24^ Qki family proteins encoding RBPs regulate several types of post-transcriptional gene regulation, including alternative splicing and mRNA stability control, during glial cell lineage.^53^ Given this expression, the Sbp2l RNA regulatory system may share some RNA targets and their regulation with the Qki family during differentiation processes but also probably generates a novel RNA regulatory network through new RNA targets and regulation in the OL cell lineage. However, the molecular function of Sbp2l, protein translation through the SECIS-like structure in the 3′UTR, would be different from those of other OL-specific RBPs, Qki, and CNPase proteins. Thus, cell-type-specific RBPs are involved in OL differentiation in different layers of RNA regulation.

Our cellular and histological analyses indicate that Sbp2l is a cytoplasmic protein but not a nuclear protein (Figures 2C and 2D). This finding suggests that the Sbp2l protein could have a similar function to the Sbp2 protein in selenoprotein synthesis. Indeed, Sbp2l possesses an RNA-binding domain, termed by the ribosomal L7Ae structure, which is similar to the Sbp2 protein. Indeed, the region of this domain showed 69.9% amino acid homology and a similar structure by alpha-fold prediction (Figures S5A–S5C). However, the other regions including the domain to catalyze incorporation of selenocysteine (Sec) into the protein in Sbp2 protein have low homology with Sbp2l, and Sbp2l is thought to lack its ability of Sec incorporation.^32^^,^^54^ Therefore, we pursued the regulation of selenoprotein synthesis in the *Gpx4* gene, a well-known target of Sbp2, to determine whether the two molecules share the same target and function. Interestingly, we found that Sbp2l lacks the activity of selenoprotein synthesis of the Gpx4 protein (Figure 3A). Our observation also would be supported by the previous analysis of zebrafish Sbp2l such that a single knockout of *Sbp2l* does not result in any reduction in selenoprotein synthesis, as shown by mass spectrometry analysis.^33^ Because this result suggests that Sbp2l has a unique role that is different from that of Sbp2, we performed transcriptome analysis to explore the unique RNA regulatory program for Sbp2l. We found that DEGs in *Sbp2l* KD OPCs are related to cholesterol biosynthesis and OL maturation (Figure 3C). In addition, IPA upstream analysis predicted Tcf7l2, a transcription factor important for cholesterol biosynthesis and OL maturation, as a top candidate of a regulator for DEGs (Figures 3E–3G).^5^ Interestingly, Tcf7l2 protein expression was significantly decreased without changes in *Tcf7l2* mRNA levels in both *in vitro Sbp2l* KD OPC and KO spinal cord (Figures 5A, 5F, S4A, and S4B). Importantly, Sbp2l binds to the 3′UTR of *Tcf7l2* mRNA and does not affect the half-life of *Tcf7l2* at either mRNA or protein levels (Figures 5B, 5C, S4D, and S4E), suggesting that Sbp2l is responsible for the translation step rather than the stabilization of *Tcf7l2* mRNA and protein through the binding to its mRNA.

In the present study, we found that Sbp2l is involved in “OL maturation” through Tcf7l2 translation using OPC/OL culture model and systemic *Sbp2l* KO mice, whereas Dai et al. revealed that Sbp2l regulates OL differentiation through Sbp2l-DIO-2-thyroid hormone T3 production pathway in *Cnp*-cre *Sbp2l* conditional KO mice.^31^ Importantly, the phenotypes of two different *Sbp2l*-deficient mice are similar to each other. Sbp2l regulates both Dio-2 and Tcf7l2 independently and mediates OL maturation.^31^ We found that the predicted downstream genes of Sbp2l are enriched in the cholesterol biosynthesis pathway, which would be an essential OL maturation pathway in lipid-enriched OLs. However, the relationship between the Sbp2l gene and human diseases by genomic analysis, such as GWAS and SNP analyses, is still unknown except for the association with lung cancer susceptibility.^55^ Our findings could help elucidate the molecular mechanisms of diseases caused by abnormal expression of Sbp2l or the disruption of Sbp2l downstream molecules. In diseases showing systemic abnormalities in lipid metabolism, e.g., Smith-Lemli-Opitz syndrome, some of their causative genes are also known to be involved in developmental disorders such as autism spectrum disorders.^56^^,^^57^ The loss of the Tcf7l2 DNA-binding transcription factor causes abnormalities in lipid metabolism and glucose tolerance in adipocytes.^58^ In addition, the variant allele of the TCF7L2 SNP is an important genetic risk factor for type 2 diabetes mellitus and is also associated with increased cardiovascular abnormalities, suggesting that impaired postprandial lipid metabolism may be associated with higher cardiovascular risk.^59^^,^^60^ In the CNS, Tcf7l2 was identified as a critical transcription factor showing dynamic expression during the remyelination process in rodent multiple sclerosis (MS), which has been thought to result from the complex features of OL damage and failure of remyelination.^40^ Indeed, the loss of Tcf7l2 results in decreased expression of the OL maturation marker genes, *Plp* and *Mbp*, suggesting the involvement of OL differentiation, although this KO mouse died shortly after birth due to systemic abnormalities, including abnormal lipid metabolism.^5^^,^^61^ Hence, given its specific expression of Sbp2l in the OL cell lineage, the roles of Sbp2l in diseases model for MS showing repeated demyelination, and remissions would also be interesting in the future. Finally, our observations add Sbp2l to a new list of OL differentiation lineage markers and suggest that Sbp2l is involved in OL differentiation via Tcf7l2 and cholesterol-biosynthesis-related genes.

### Limitations of the study

In this study, we proposed a new mechanistic insight of oligodendrocyte (OL) maturation through translational control of Tcf7l2 transcriptional factor by an OL-specific RBP, Sbp2l. There are several limitations to this study that must be acknowledged. First, previously identified list of selenoproteins did not include Tcf7l2, and it is not possible to exclude that Tcf7l2 protein contains selenocysteine. Second, we used IP-qPCR method to identify the interaction of Sbp2l protein and *Tcf7l2* mRNA. Further studies deciphering the Sbp2l binding in transcriptome-wide manner using HITS-CLIP analysis may clarify the roles in OL maturation.

## STAR★Methods

### Key resources table

**Table:** 

REAGENT or RESOURCE	SOURCE	IDENTIFIER
**Antibodies**		

Rabbit polyclonal anti-Sbp2l, IHC/ICC: 1:500, WB 1:1000	This paper, see methods details-antibody production	N/A
Mouse monoclonal anti-Qki7, clone N183/15, IHC/ICC: 1:200	UC Davis/NIH NeuroMab Facility	Cat# N183/15; RRID:AB_2877379
Mouse monoclonal anti-Olig2, clone 211F1.1, IHC/ICC: 1:250	Sigma-Aldrich	Cat# MABN50; RRID:AB_10807410
Rabbit polyclonal anti-PDGFRα, IHC/ICC: 1:500	Cell Signaling Technology	Cat# 3164; RRID:AB_2162351
Rat monoclonal anti-PDGFRα, APA5, IHC: 1:500	Thermo Fisher Scientific	Cat# 67-1401-80, RRID:AB_2717164
Mouse monoclonal anti-MBP, WB: 1:5000	Abcam	Cat# ab62631; RRID:AB_956157
Mouse monoclonal anti-MBP, clone QD-9, IHC: 1:200	Abcam	Cat# ab22460, RRID:AB_447071
Rat monoclonal anti-MBP, IHC/ICC: 1:1000	Millipore	Cat# MAB386; RRID:AB_94975
Mouse monoclonal anti-PLP, IHC: 1:1000	Bio-Rad	Cat# MCA839G; RRID:AB_2237198
Mouse monoclonal anti-Glutamine Synthetase, IHC: 1:400	BD Biosciences	Cat# 610518; RRID:AB_397880
Mouse monoclonal anti-GFAP, clone G-A-5, IHC: 1:500	Sigma-Aldrich	Cat# G3893; RRID:AB_477010
Mouse monoclonal anti-Actin, clone C4, WB: 1:5000	Millipore	Cat# MAB1501; RRID:AB_2223041
Mouse monoclonal anti-GAPDH, clone 6C5, WB: 1:1000	Abcam	Cat# ab8245; RRID:AB_2107448
Rabbit polyclonal anti-Sbp2, WB: 1:1000	Proteintech	Cat# 12798-1-AP; RRID:AB_2186555
Rabbit polyclonal anti-Gpx4, clone EPNCIR144, WB: 1:1000	Abcam	Cat# ab125066; RRID:AB_10973901
Mouse monoclonal anti-α-Tubulin, clone DM1A, WB: 1:5000	Sigma-Aldrich	Cat# T9026; RRID:AB_477593
Rabbit monoclonal anti-Tcf7l2, clone C48H11, IHC: 1:2000 and WB: 1:1000	Cell Signaling Technology	Cat# 2569; RRID:AB_2199816
Mouse monoclonal anti-Tcf7l2, clone D-4, IHC/ICC: 1:200	Santa Cruz Biotechnology	Cat# sc-166699; RRID:AB_2199823
Normal Rabbit IgG	Millipore	Cat# 12-370
Donkey anti-rabbit IgG (H + L) Alexa Fluor 555	Thermo Fisher Scientific	Cat# A-31572 RRID:AB_162543
Goat anti-rabbit IgG (H + L) Alexa Fluor 488	Thermo Fisher Scientific	Cat# A-11034 RRID:AB_2576217
Goat anti-mouse IgG (H + L) Alexa Fluor 555	Thermo Fisher Scientific	Cat# A-11029 RRID:AB_2534088
Goat anti-mouse IgG (H + L) Alexa Fluor 488	Thermo Fisher Scientific	Cat# A-21424 RRID: AB_141780
Goat anti-rat IgG (H + L) Alexa Fluor 555	Thermo Fisher Scientific	Cat# A-21434 RRID: AB_2535855
Goat Anti-Rabbit IgG(H + L), Mouse/Human ads-HRP	SouthernBiotech	Cat# 4050-05, RRID:AB_2795955
Goat Anti-Mouse IgG(H + L), Human ads-HRP	SouthernBiotech	Cat# 1031-05, RRID:AB_2794307
Rat monoclonal Anti- PDGFRa(CD140a), Unconjugated, Clone APA5, for a panning of OPCs	BD Biosciences	Cat# 558774, RRID:AB_397117

**Chemicals, peptides, and recombinant proteins**		

Lipofectamine RNAi MAX	Thermo Fisher Scientific	Cat# 13778150
CHX	Sigma-Aldrich	Cat# C4859
Papain, PAP2	Worthington biochemical	Cat# LK003178
DNase I, DCLS	Worthington biochemical	Cat# LS002060
Ovomucoid protease inhibitor, OI-BSA	Worthington biochemical	Cat# LK003182
Bandeiraea Simplicifolia Lectin I (BSL 1)	Vector laboratories	Cat# L-1100-5
TrypZean	Sigma-Aldrich	Cat# T3568
Poly-D-lysine	Sigma-Aldrich	Cat# P6407
TrypLE select	Thermo Fisher Scientific	Cat# 12563011
Insulin	Sigma-Aldrich	Cat# I6634
Forskolin	Sigma-Aldrich	Cat# F6886
thyroid hormone T3	Sigma-Aldrich	Cat# T6397
CNTF	PeproTech	Cat# AF450-13
NT-3	PeproTech	Cat# 450-03
PDGF-AA	PeproTech	Cat# AF100-13A
d-Biotin	Sigma-Aldrich	Cat# B4639
N-acetyl-L-cysteine	Sigma-Aldrich	Cat# A8199
Progesterone	Sigma-Aldrich	Cat# P8783
Sodium selenite	Sigma-Aldrich	Cat# S5261
BSA	Sigma-Aldrich	Cat# A4161
Transferrin	Sigma-Aldrich	Cat# T1147
Putrescine	Sigma-Aldrich	Cat# P5780
Antibiotics	Thermo Fisher Scientific	Cat# 15140122
Glutamax	Thermo Fisher Scientific	Cat# 35050061
pyruvate	Thermo Fisher Scientific	Cat# 11360070
Trace element B	Corning	Cat# 25-022-CI
N21-MAX Media	R&D Systems	Cat# AR008
Opti-MEM I reduced serum medium	Thermo Fisher Scientific	Cat# 31985062
FluoroMyelin Green Fluorescent Myelin Stain	Thermo Fisher Scientific	Cat# F34651
Hoechst 33258	Sigma-Aldrich	Cat# B2883
RIPA Buffer	Cell Signaling Technology	Cat# 9806

**Critical commercial assays**		

DC Protein Assay Kit II	Bio-Rad	Cat# 5000122
Can Get Signal Solution I	TOYOBO	Cat# NKB-201
ECL Western Blotting Substrate	Thermo Fisher Scientific	Cat# 32106
RNeasy Micro kit	Qiagen	Cat# 74004
RNeasy 96 kit	Qiagen	Cat# 74181
High-Capacity cDNA Reverse Transcription Kit	Thermo Fisher Scientific	Cat# 4368814
TaqMan™ Gene Expression Master Mix	hermo Fisher Scientific	Cat# 4369016
TruSeq Stranded mRNA Library Prep for NeoPrep kit	Illumina	Cat# NP-202-1001
Click-iT Nascent RNA Capture Kit	Thermo Fisher Scientific	Cat# C10365
Zenon™ Rabbit IgG Labeling Kits	Thermo Fisher Scientific	Cat# Z25305

**Deposited data**		

Dataset for mouse OPC differentiation series	This paper	GEO: GSE228241
Dataset for mouse OPC Sbp2l KD series	This paper	GEO: GSE228245
Mouse brain RNA-seq	Zhang et al.^25^	GEO: GSE52564
Mouse brain single cell RNA-seq	Zeisel et al.^26^	GEO: GSE60361
Human brain RNA-seq	Zhang et al.^27^	GEO: GSE73721
Human whole tissue pSI values (25 tissues from GTEx dataset)	Wells et al.^28^	https://academic.oup.com/nar/article/43/22/10804/1804831 See Table S3 for pSI values

**Experimental models: Cell lines**		

Hela Tet-On 3G cell line	Clontech	Cat#631183; RRID:CVCL_V353

**Experimental models: Organisms/strains**		

Sbp2l knock-out mice	This paper, see methods details-mice	N/A
Cultured mouse oligodendrocyte precursor cells	This paper, see methods details-mouse OPC culture	N/A

**Oligonucleotides**		

guide RNA	This paper, see methods details-mice	N/A
single strand oligodeoxynucleotide	This paper, see methods details-mice	N/A
Primers for qRT-PCR and Genotyping PCR	This paper, see Table S3	N/A
Sbp2 siRNA (siRNA ID:s93475, s93476, s93477)	Thermo Fisher	Cat#4390771
Sbp2l siRNA (siRNA ID:s88726, s88727, s88728)	Thermo Fisher	Cat#4390771

**Recombinant DNA**		

Sbp2l_pTracer-SV40	This paper, see supplementary methods	N/A
3xFLAG_Tcf7l2_pRP-neo-SV40	This paper, see supplementary methods	NM_001331146.1 (VB230223-1612ehv)
pTRE-Tight-BI-NLS-Venus-PEST-NLS-tdTomato-3PEST (pDual_FluR)	Nogami et al.^39^	N/A
SECIS-like#1_in_pDual_FluR	This paper, see supplementary methods	N/A
SECIS-like#2_in_pDual_FluR	This paper, see supplementary methods	N/A

**Software and algorithms**		

pSI (version 1.1)	Dougherty et al.^22^	https://www.rdocumentation.org/packages/pSI/versions/1.1/topics/specificity.index
OmicSoft ArrayStudio	Qiagen	N/A
DESeq2	https://bioconductor.org/packages/release/bioc/html/DESeq2.html	RRID:SCR_015687
QIAGEN Ingenuity Pathway Analysis	Qiagen	RRID:SCR_008653
RNAstructure	https://rna.urmc.rochester.edu/RNAstructureWeb/Servers/Predict1/Predict1.html	RRID:SCR_017216
AlphaFold Protein Structure Database	https://alphafold.ebi.ac.uk/	RRID:SCR_023662
R (Version 4.1.2)	http://www.r-project.org/	RRID:SCR_001905
ImageJ	https://ImageJ.net/	RRID:SCR_003070

**Other**		

FV1200	Olympus	N/A
BZ-X810	Keyence	N/A
Hybrid Cell Count Module BZ-H4C	Keyence	N/A
MiSeq system	Illumina	N/A
HiSeq 2500 system	Illumina	N/A
Viia 7real-time PCR detection system	Thermo Fisher Scientific	N/A
StepOnePlus	Thermo Fisher Scientific	N/A
ImageQuant LAS-4000	Fujifilm	N/A
iBright Imaging Systems	Thermo Fisher Scientific	N/A

### Resource availability

#### Lead contact

Further information and requests for resources and reagents should be directed to and will fulfilled by the lead contact, Dr. Masato Yano (myano@med.niigata-u.ac.jp).

#### Material availability

All unique/stable reagents generated in this study are available from the lead contact without restriction.

#### Data and code availability

• The datasets generated and analyzed in the present study were deposited in the NCBI Gene Expression Omnibus (GEO) repository under accession numbers GSE228241 (for OPC differentiation series) and GSE228245 (for OPC KD series). These accession numbers are also listed in the key resources table.
• This paper does not report original code.
• Any additional information required to reanalyze the data reported in this paper is available from the lead contact upon request.

### Experimental model and study participant details

#### Cell lines

HeLa Tet-On 3G cell line (Clontech, Cat#631183) was obtained and cultured as directed by the supplier.

### Methods details

#### Screening for OLs-specific RBPs

The pSI package^22^ with three human and mouse brain datasets^25^^,^^26^^,^^27^ were used to identify OL-specific RBPs for both humans and mice. The specificity for the nervus system among whole tissues was considered by GTEx data.^28^ Fourteen genes were identified as OL-specific genes by merging significant genes in four datasets (pSI <0.05). Among them, three RBPs, *Cnp*, *Sbp2l*, and *Larp6*, were identified as the potential OL-specific RBPs by referring to the 1,542 RBPs.^11^

#### Mice

All procedures were performed in accordance with the standards of humane care, and the treatment of the research animals was approved by the Institutional Animal Care and Use Committee (IACUC) in Takeda Pharmaceutical Company Ltd.

For generation of *Sbp2l* null mice, we used 5′- and 3′- guide RNA (gRNA) and single strand oligodeoxynucleotide (ssODN) sequences as below.

5′-gRNA target sequences: AGTCCAGGACGAGATGCCAT.

3′-gRNA target sequences: GAGTGAGTTAGTGACTCGA

ssODN sequences:

CAGCATTTGGTTTATAGCTTTAGAACCATAATAATTAGGAGTTCAGTACTACTTCCGATGCGAAGGAATGGATCAGAGTTTCTTGTTGAATATTTTCTCCTCTTCCCTTATGTCCTTTAG

Homozygous null mice were selected by qPCR detection for *Sbp2l* intron15 sequence.

#### Isolation of mouse oligodendrocyte precursor cells (OPCs)

OPCs were purified by immunopanning as essentially described in Emery and Dugas (2013).^62^ Briefly, cortices were dissected from three pups of postnatal day 7 mice with C57BL/6J genetic background and treated with papain (Worthington biochemical, PAP2, 20 units/mL) and DNase I (Worthington biochemical, DCLS, 125 units/mL) in high glucose EBSS buffer with calcium and magnesium. After incubation at 34°C for 90 min, cortices were rinsed three times with 0.1% ovomucoid protease inhibitor (Worthington biochemical, OI-BSA) supplemented with DNase (250 units/mL) and dissociated successively with a 10-mL pipette, a 5-mL pipette, and a 1-mL pipette tip. The cell suspension was layered on 0.5% ovomucoid inhibitor and pelleted at 220 × g for 6 min. The cell pellet was resuspended with 10 mL of 0.02% BSA (Sigma-Aldrich) in D-PBS with calcium and magnesium with DNase (125 units/mL) and insulin (Sigma-Aldrich, 5 μg/mL) and incubated successively on two BSL I-coated negative panning plates (Vector laboratories, 5 μg/mL) to remove microglia and endothelial cells. The cell suspension was then positively selected with a panning plate coated with a monoclonal antibody against PDGFRα (BD Pharmingen 558774, 20 μg per dish). Bound cells were detached with TrypZean (Sigma-Aldrich). Approximately 3 × 10^5^ cells were obtained and plated onto a poly-D-lysine-coated 10-cm tissue culture dish with OPC growth media containing DMEM, antibiotics, 1 × sodium pyruvate (Thermo Fisher Scientific), 1 × Glutamax I (Thermo Fisher Scientific), 0.1 ×x Trace Elements B (Corning), 1 × N21-MAX Media Supplement (R&D Systems, AR008), 5 μg/mL insulin (Sigma-Aldrich), 5 μg/mL N-acetyl cysteine (Sigma-Aldrich), 100 μg/mL BSA, 100 μg/mL apo-transferrin (Sigma-Aldrich), 16 μg/mL putrescine (Sigma-Aldrich), 60 ng/mL progesterone (Sigma-Aldrich), 40 ng/mL sodium selenite (Sigma-Aldrich), 10 ng/mL d-Biotin (Sigma-Aldrich), 10 ng/mL PDGF-AA (Peprotech), and 10 ng/mL CNTF (Peprotech). OPCs were differentiated following removal of PDGF-AA and addition of thyroid hormone T3 (Sigma-Aldrich 40 ng/mL).

#### siRNA transfection

Primary OPCs were seeded at a density of 1.5 × 10^5^ cells/well in 6-well PDL-coated plates (Iwaki). After a 24 h incubation, the cells were transfected with 20 nM Silencer Select siRNAs using Opti-MEM I reduced serum medium (Thermo Fisher Scientific) and Lipofectamine RNAi Max (Life Technologies), according to the manufacturer’s instructions, and then were incubated at 37°C for 72 h. The cells were harvested and subjected to the mRNA and protein analyses including RNA-seq library generation.

#### Antibody production

For Sbp2l-specific antibodies, rabbits were independently immunized with the recombinant protein (amino acids 466–1089) and KLH-conjugated C-terminal peptide (amino acids 1021–1089) of Sbp2l. The antibodies were affinity purified from rabbit serum by using each recombinant or peptide of Sbp2l. Their specificity was confirmed by Western blots and immunohistochemistry in *Sbp2l* KD OPC cells and *Sbp2l* KO spinal cord tissue, respectively (Figures S1A and 4B).

#### Tissue preparation and immunostaining

The mouse spinal cord was fixed with 4% paraformaldehyde in phosphate-buffered saline (PBS) and postfixed in the same fixative. The samples were then cryoprotected with 30% sucrose/PBS and sectioned with a cryostat (18-μm sections) (CM1850UV, Leica). The tissue was then subjected to immunohistochemistry (IHC). The sections were boiled in 10 mM citric acid buffer (pH 6.0) for 5 min before blocking for all antibodies except for the anti-MBP and anti-PLP antibodies for antigen retrieval. For immunostaining for MBP and PLP, sections were treated with ice-cold MeOH for 6 min before performing immunofluorescence staining. Then, the sections were incubated overnight at 4°C with primary antibodies (see the supplemental information for Antibodies) followed by incubation with Alexa-dye conjugated secondary antibodies (Invitrogen 1:1000). For FluoroMyelin assay, the sections were incubated for 20 min at room temperature with FluoroMyelin (1:300 F34651, Thermo Fisher) and counterstained with Hoechst dye. Images of the immunostained specimens were collected using a confocal laser scanning microscope (FV1200: Olympus or BZ-X810: Keyence).

#### mRNA sequence

Total RNAs were obtained from two independent replicates of cultured OPCs. The RNAs were extracted using RNeasy Micro kit (Qiagen) followed by the generation of stranded mRNA-seq libraries using TruSeq Stranded mRNA Library Prep for NeoPrep kit (Illumina) on the NeoPrep Library Prep System. The multiplexed libraries were sequenced as 75-nt pair-end runs on a MiSeq system and 100-nt on HiSeq 2500 system for OPC differentiation and OPC KD series, respectively. Fastq files were processed by OmicSoft ArrayStudio (Qiagen), differentially expressed genes (DEGs) were identified by the DESeq2 method. Following pathway analysis was performed using ingenuity pathway analysis (IPA) software (Qiagen).

#### CLIP-qPCR

CLIP using homemade anti-Sbp2l-specific antibodies (see above) was performed using UV-irradiated mOLs.^35^ Immunoprecipitated RNA-protein complex were digested by Proteinase K, then RNAs were extracted using phenol/chloroform and precipitated by ethanol precipitation method. Isolated RNAs were solved in RNase-free water, and then subjected to qRT-PCR assay. Normal rabbit IgG (12–370, Merck millipore) was used as a negative control.

#### Quantitative RT-PCR (qRT-PCR) assay

Total RNAs were extracted from siRNA-transfected OPC cells using an RNeasy 96 kit (QIAGEN). The cDNA from 25 ng of total RNA was synthesized using a High-Capacity cDNA Reverse Transcription Kit (Thermo Fisher Scientific) according to the manufacturer’s instructions. The amount of each mRNA was quantified using a TaqMan assay with TaqMan Gene Expression Master Mix (Thermo Fisher Scientific) and gene-specific probe and primers synthesized by Sigma Genosys (sequences are listed in Table S3). At least three biological replicates were used for quantitative PCR, which was performed with the Viia7 real-time PCR detection system and StepOnePlus real-time PCR system (Thermo Fisher Scientific).

#### Western blotting

siRNA-transfected OPC cells were lysed with RIPA Buffer (Cell Signaling Technology). The lysates were quantified by DC Protein Assay Kit II (BIO-RAD), and then 5 μg of the lysates separated by electrophoresis in a 7.5–15% polyacrylamide gel (DRC) and blotted onto a PVDF membrane (Millipore). The membrane was blocked with 3% BSA in TBS, incubated with primary antibody diluted in Can Get Signal Solution I (TOYOBO) overnight, incubated with HRP-linked anti-mouse or anti-rabbit IgG diluted in TBS-T for 1 h, and developed with ECL (GE Healthcare). Images were visualized using an ImageQuant LAS-4000 imaging system (Fujifilm) or iBright Imaging Systems (Thermo Fisher Scientific).

#### Pulse-chase assay for *Tcf7l2* mRNA

Pulse labeling was performed using Click-iT Nascent RNA Capture Kit (Thermo Fisher) according to a manufacturer’s protocol. Briefly, OPCs at 3 days post siRNA transfection were incubated in EU containing medium for 0.5 h, and then washed with D-PBS. The cells were further incubated for 0, 2 and 6 h, and harvested. The following labeled RNA isolation was performed with Click-iT Nascent RNA Capture Kit and subjected to qRT-PCR assay.

#### Cycloheximide (CHX) chase assay for Tcf7l2 protein

Tcf7l2 protein degradation was analyzed by CHX chase assay in the primary OPC cells. OPC cells were transfected with control and *siSbp2l* by RNAi Max. Three days after transfection, 30 μg/mL CHX were added to inhibit protein synthesis. The cells were collected at 0, 3, 6 h after the treatment with CHX, followed by Western blotting with anti-Tcf7l2 and anti-α-Tubulin antibodies. The Tcf7l2 degradation rate was analyzed as a ratio of protein remaining relative to α-Tubulin protein by ImageJ between control and si*Sbp2l*.

#### Assay for Sbp2l knock down with Tcf7l2 overexpression

*Sbp2l* siRNA was transfected into cultured OPCs using RNAi MAX (Thermo Fisher) and Tcf7l2 expression or control vector were transfected with lipofectamine 3000 at 24 h post-siRNA transfection according to a manufacturer’s protocol (Thermo Fisher). Total RNAs were collected 48 h post-transfection. RNA expression levels were analyzed by qRT-PCR assay.

#### Vector construction and plasmid preparation

Mouse Sbp2l cDNA were subcloned into the *Kpn* I/*Not* I site of pTravcer-SV40 with PA:DYK tag into *Kpn* I/*Spe* I. Dual color reporters were engineered based on the bidirectional tetracycline-inducible vector, pTRE-Tight-BI with NLS-tdTomato-PEST and NLS-Venus-PEST as previously reported.^39^ SECIS-like elements were subcloned into the *Eco*R V site in MCS I of the pTRE-Tight-BI vector. The detailed sequences are shown as below. Tcf7l2 expression vector containing synthesized 3xFLAG/Tcf7l2 (NM_001331146.1) under the SV40 promotor in pRP-neo (VB230223-1612ehv) was constructed by Vectorbuilder.

>Secis-like element #1

CGCCGTGGCTACATGAGTTAATGTTTATGGAGTTCATTGGTCAATATTTGACCCATTCTTATTTCAATTTCTCCTTTTAAATATGTAGATGAGAAAAACCTCATGATTCTACCAAAA

>Secis-like element #2

AGAAAGCATTTTAAATGAAGAGGTCTAAACCCTTAAGGGCCAAAAAAAATCCTGTATATTAGATTACTCTTAAATGAAAAAGAAAA

#### Measurement of live-cell dual color reporter assay

HeLa Tet-On 3G cells were cultured in DMEM (WAKO) with10% Fetal bovine serum medium. For transfection of the expression vectors, ratiometric fluorescence reporter in pTRE-Tight-BI with or without SECIS-like fragment, 5×10^4^ cells were seeded in 24-well plates, or 2.5x10^4^ cells were seeded in 48-well plates (Thermo Fisher). Seven hours after transfection, the culture medium containing transfection reagents was changed to fresh medium containing 10 ng/mL doxycycline. Twenty-four hours after transfection, Images of Venus (50ms) and tdTomato (5ms) were obtained from three fields of view for each well by the KEYENCE BZ-X810. Nuclei with Venus and tdTomato were automatically selected and the sum of the Venus and tdTomato signals in each well was calculated from the Venus and tdTomato signal intensities of each cell in the well by Hybrid Cell Count Module BZ-H4C (KEYENCE). The relative sum of the tdTomato signal intensity to the sum of the Venus signal intensity in each well was calculated as the translational activity and relative values are shown in the graph. Quantification of Venus and tdTomato fluorescence signal from single cells were automatically obtained and quantified using the Hybrid Cell Count Module BZ-H4C (KEYENCE).

#### Bioinformatic tools

##### Prediction of RNA secondary structure

RNAstructure, a web-based RNA secondary structure prediction tool, was used for the analysis for of RNA secondary structure.

https://rna.urmc.rochester.edu/RNAstructureWeb/Servers/Predict1/Predict1.html.

##### IPA canonical pathway and upstream analyses

Those pathway analyses were performed by Ingenuity Pathway Analysis software (QIAGEN).

##### Prediction of protein structure

The AlphaFold Protein Structure Database (DeepMind) was used for the accurate prediction of protein structures.

https://alphafold.ebi.ac.uk.

### Quantification and statistical analysis

#### Statistical analysis

All data analyses were conducted using R (Version 4.1.2). Mann-Whitney *U* test was employed for comparisons between two groups, WT and KO to analyze Tcf7l2 expression level. To analyze RNA and protein level expression, Welch’s *t* test, Dunnett’s test and Two-tailed Student’s *t* test were utilized for inter-group comparisons; differences of at least p < 0.05 were statistically significant.

## Supplemental information

Document S1. Figures S1–S5, Tables S1, and S2

Table S3. Oligonucleotides used in the study, related to STAR Methods

## Footnotes

Supplemental information can be found online at https://doi.org/10.1016/j.isci.2023.108451.
