# Supplementary material for: Sbp2l contributes to oligodendrocyte maturation through translational control in Tcf7l2 signaling
Source: iScience. 2023 Nov 16;26(12):108451. doi: 10.1016/j.isci.2023.108451 (PMC10783607; doi:10.1016/j.isci.2023.108451)
Supplement: Document S1. Figures S1–S5, Tables S1, and S2 [file mmc1.pdf]

iScience, Volume *26*

Supplemental information

Sbp2l contributes to oligodendrocyte maturation through translational control in Tcf7l2 signaling

Masato Yugami, Yoshika Hayakawa-Yano, Takahisa Ogasawara, Kazumasa Yokoyama, Takako Furukawa, Hiroe Hara, Kentaro Hashikami, Isamu Tsuji, Hirohide Takebayashi, Shinsuke Araki, Hideyuki Okano, and Masato Yano

Table S1. Gene list identified by the bioinformatic screening system using cell-type specificity index (pSI), related to Figure 1.

	Human Brain RNA-seq	Mouse brain RNA-seq.			Mouse brain scRNA-seq.						25 Tissues RNA-seq.	
Symbol_M	Oligo	OPC	Newly_OL	Myelin_OL	Oligo1	Oligo2	Oligo3	Oligo4	Oligo5	Oligo6	Brain	Nerve
Cnp	0.0024	1.0000	0.0255	0.0015	0.0066	0.0068	0.0327	0.0035	0.0143	0.0180	0.0113	0.0075
Fez1	0.0132	1.0000	1.0000	0.0052	1.0000	0.0417	1.0000	0.0643	0.0148	0.0367	0.0020	0.0117
Kif13b	0.0223	1.0000	1.0000	0.0269	1.0000	1.0000	0.0103	0.0707	0.0257	0.0234	1.0000	0.0417
Larp6	0.0064	1.0000	0.0735	0.0027	1.0000	0.0491	1.0000	1.0000	0.0603	1.0000	0.0486	1.0000
Mbp	0.0022	1.0000	0.0301	0.0003	0.0029	0.0115	0.0110	0.0066	0.0160	0.0260	0.0000	0.0031
Ndrg1	0.0091	1.0000	0.0854	0.0014	1.0000	0.0133	0.0100	0.0631	0.0083	0.0114	1.0000	0.0098
Nipa1	0.0248	1.0000	0.0572	0.0358	0.0104	0.0207	0.0742	0.0175	0.0217	0.0483	0.0144	1.0000
Olig2	0.0034	0.0135	0.0551	1.0000	0.0028	0.0054	0.0282	0.0135	0.0364	0.0294	0.0000	1.0000
Otud7b	0.0124	1.0000	0.0802	0.0435	0.0272	0.0166	0.0838	0.0543	0.0275	0.0565	1.0000	0.0136
Prrg1	0.0115	1.0000	1.0000	0.0185	1.0000	0.0154	0.0058	1.0000	0.0064	0.0133	0.0446	0.0486
Ptpdc1	0.0167	1.0000	0.0635	0.0163	0.0693	0.0100	0.0627	0.0670	1.0000	1.0000	1.0000	0.0042
Sbp2l	0.0428	1.0000	0.0367	0.0326	0.0178	0.0142	0.0427	0.0831	0.0361	0.0807	0.0765	0.0032
Synj2	0.0119	1.0000	0.0937	0.0041	1.0000	0.0361	0.0906	0.0549	0.0443	1.0000	0.0288	1.0000
Trim59	0.0084	1.0000	0.0412	0.0094	0.0446	0.0047	0.0314	0.0502	0.0145	0.0099	0.0063	1.0000

Table S2. The differentially expressed genes (DEGs) between control and Sbp2l KD mOPCs identified by RNA-seq analysis, related to Figure 3.

Rank	Gene Name	Mean FC	Max FPKM
1	*H19*	6.01	1314.16
2	*Irs4*	5.79	18.53
3	*Slitrk1*	5.21	15.18
4	*Erbb4*	4.79	65.71
5	*Myo1b*	4.48	11.70
6	*Tox*	4.48	31.01
7	*Rftn2*	4.31	18.05
8	*Rgcc*	4.25	62.78
9	*Dcx*	4.24	23.80
10	*Itga8*	4.14	37.39
11	*Efhd1*	4.07	23.90
12	*Glipr2*	4.00	24.17
13	*Ets1*	3.99	25.89
14	*Gpr179*	3.97	10.35
15	*Aif1l*	3.84	42.68
16	*Ephb3*	3.82	12.11
17	*Gpr125*	3.81	56.80
18	*Tox3*	3.77	24.73
19	*Flna*	3.70	89.34
20	*Adamts1*	3.54	88.48
⋮			
418	*Lamp1*	-8.87	839.07
419	*Fah*	-9.22	14.18
420	*Ddc*	-9.53	50.57
421	*Cryab*	-9.81	22.83
422	*Renbp*	-9.97	10.55
423	*1700047M11Rik*	-10.16	20.56
424	*Pdlim2*	-10.19	75.97
425	*Sema4a*	-10.59	30.84
426	*Plau*	-10.81	48.83
427	*Sept4*	-13.45	173.76
428	*Ppfibp2*	-13.74	46.73
429	*Tmem125*	-13.91	12.43
430	*Ptpru*	-14.57	16.42
431	*Tmem151a*	-15.74	30.63
432	*1300002K09Rik*	-18.90	12.13
433	*Tspan2*	-19.80	174.80
434	*Rnf112*	-26.30	20.11
435	*Pmp2*	-30.53	32.13
436	*Alox5*	-41.64	24.45
437	*Sbp2l*	-52.56	255.22

Comparisons between control and *Sbp2l* KD mOPCs were performed independently for each dataset and only genes with |fold change| ≧ 2, FDR < 0.05, and FPKM ≧ 10 (at least 1 sample) were considered as common *Sbp2l* KD DEGs. We identified 437 common DEGs (77 upregulated and 360 downregulated genes in *Sbp2l* KD mOPCs) between these cells. Table represents top 20 (upregulated) and bottom 20 (downregulated) DEGs.

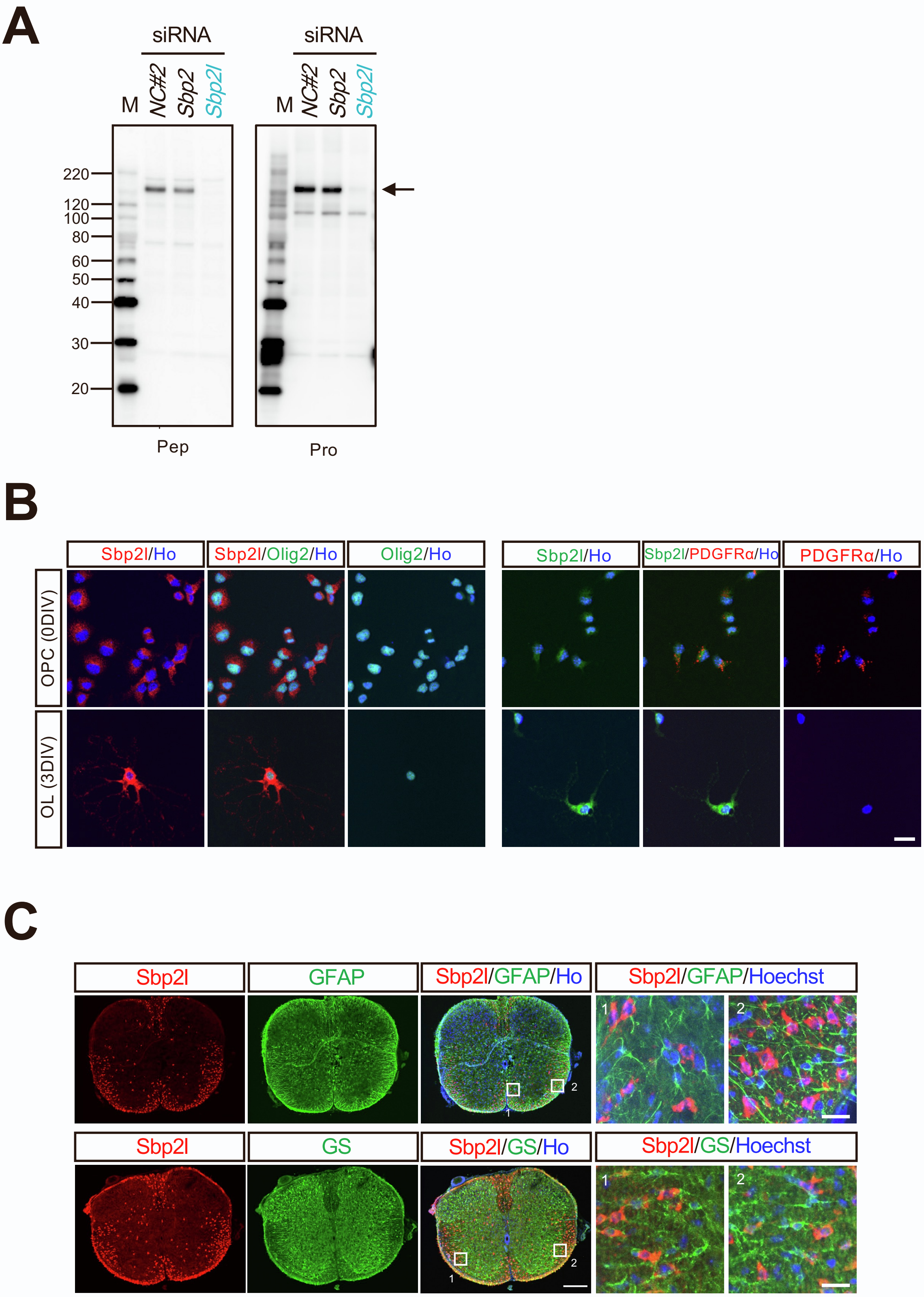

A
siRNA
M
NC#2
Sbp2
Sbp2l
220
120
100
80
60
50
40
30
20
Pep
Pro
B
Sbp2l/Ho
Sbp2l/Olig2/Ho
Olig2/Ho
Sbp2l/PDGFRα/Ho
PDGFRα/Ho
OPC (0DIV)
OL (3DIV)
C
Sbp2l
GFAP
Sbp2l/GFAP/Ho
Sbp2l/GFAP/Hoechst
GS
Sbp2l/GS/Ho
Sbp2l/GS/Hoechst
1
2

Figure S1. Verification of generated anti-Sbp2l antibodies and Sbp2l protein expression, related to Figure 2.

A, Western blots for validation of originally generated two different anti-Sbp2l antibodies using control or *Sbp2l* KD OPCs. Note that anti-Sbp2l antibodies recognized the main band indicated by an arrow in the control (NC#2) and *siSbp2* condition, which were clearly lost in *Sbp2l* KD condition. Pep: anti-peptide antibody, Pro: anti-recombinant protein antibody.

B, Immunocytochemistry using antibodies against Sbp2l (red) and Olig2 (green) (left panel) and Sbp2l (green) and PDGFRα (red) (right panel) combined with Hoechst staining to detect nuclei. The representative pictures at 0 day (OPC 0DIV, top) or 3 days (OL 3DIV, bottom) after induction of OL differentiation. Scale bar indicates 20 µm.

C, Immunohistochemistry using P7 mouse spinal cord sections indicating that Sbp2l protein (red) is not detectable in GFAP-positive or Glutamine synthetase (GS)-positive astrocytes (green). Enlarged view indicated by insets were shown in the right two panels. Scale bars, 200 µm for low magnification and 20 µm for higher magnification.

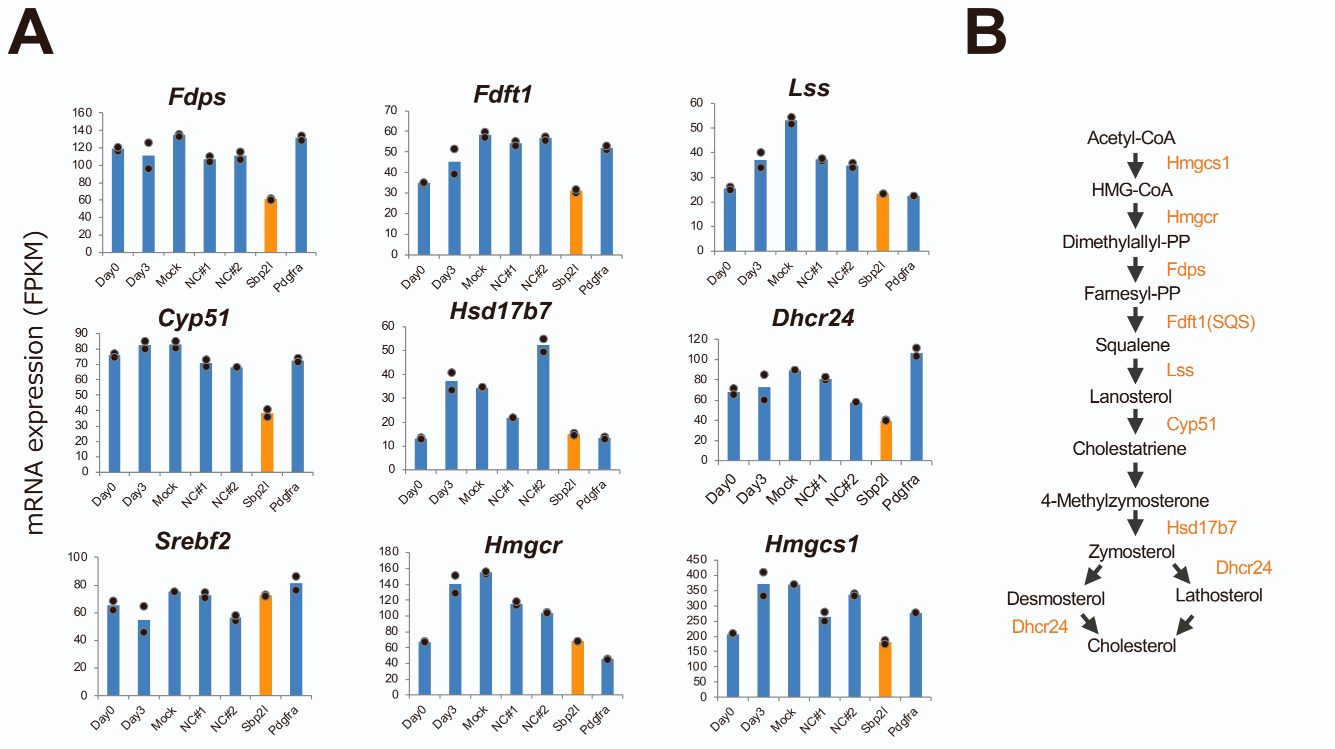

Figure S2. *Sbp2l* KD affected to the cholesterol pathway-related genes, related to Figure 3.
A, RNA-seq analysis revealed that decreased mRNA expression levels (FPKM) of the cholesterol biosynthesis-related genes in *Sbp2l* KD mOPCs. Bar graphs represent means. Each dot represents independent experiment.
B, Illustration of cholesterol biosynthesis pathway with downregulated DEGs in *Sbp2l* KD mOPCs high-lightened by orange color.

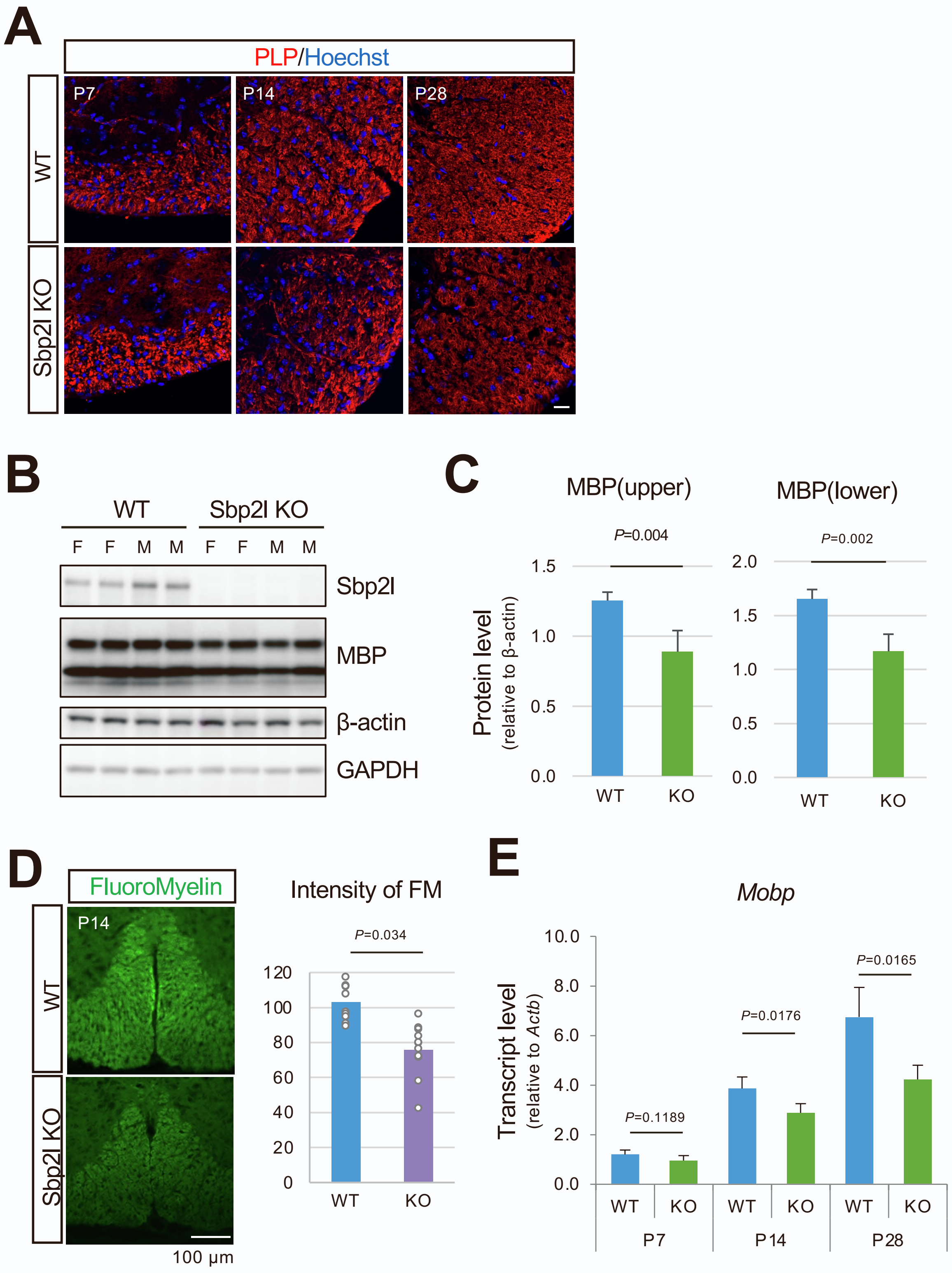
A
PLP/Hoechst
P7
P14
P28
WT
Sbp2l KO
B
WT
Sbp2l KO
F F M M F F M M
Sbp2l
MBP
β-actin
GAPDH
C
MBP(upper)
MBP(lower)
P=0.004
P=0.002
Protein level
(relative to β-actin)
WT
KO
D
FluoroMyelin
P14
WT
Sbp2l KO
100 µm
Intensity of FM
P=0.034
WT
KO
E
Mobp
Transcript level
(relative to Actb)
P=0.1189
P=0.0176
P=0.0165
WT KO WT KO WT KO
P7
P14
P28

Figure S3. ***Sbp2l*** **KO affected to OLs maturation, related to Figure 4.**
A, PLP protein expression. Immunohistochemistry for PLP protein in WT and *Sbp2l* KO spinal cords at the developing age. Scale bars, 20 μm.
B, Western blots revealed that the decreased MBP expression in adult *Sbp2l* KO.
C, Quantification for Western blots. Densitometric analysis was performed using Image J. MBP protein signal was normalized to β-actin. Bar graphs indicated that the significant decrease of MBP protein in *Sbp2l* KO spinal cord. Data represent the mean ± SD of four independent experiments. Two-tailed student's *t* test.
D, Myelin assay in P14 wild-type and *Sbp2l* KO spinal cord. To examine the myelin abundance, mouse sections were stained with FluoroMyelin (FM) (left). Signal intensity of FM was analyzed with Image J. Bar graph shows the intensity of FM relative to background signa (right). Data represent the mean of three independent experiments. Each dot represents a fluorescence signal for the FM. Two-tailed student's *t* test.
E, qRT-PCR analysis shows the decrease of *Mobp* mRNA in the spinal cord tissues of *Sbp2l* KO compared to those of WT mice. Data represent the mean ± SD of four independent experiments. Welch's *t* test.

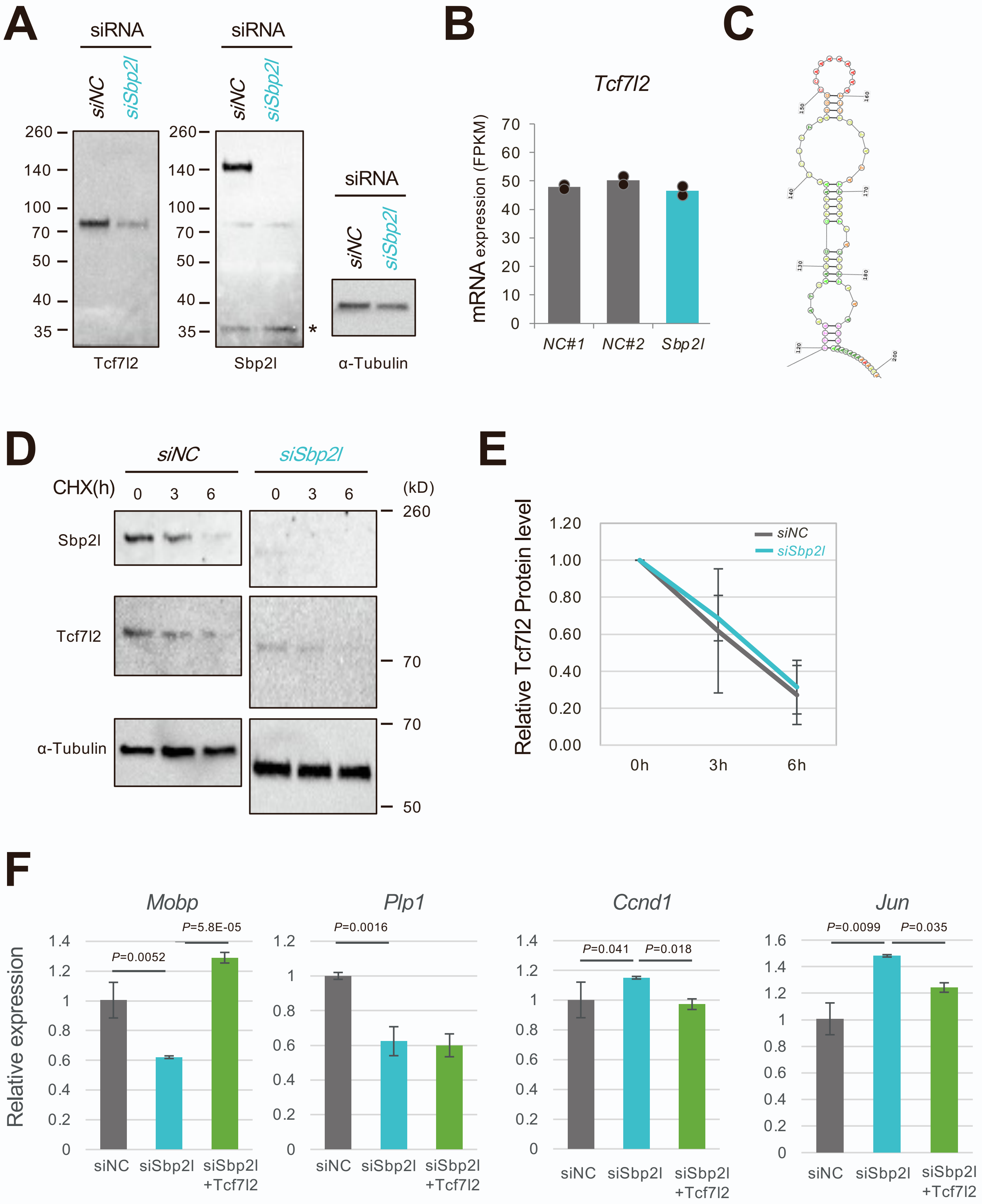

A
siRNA
siNC
siSbp2l
260
140
100
70
50
40
35
Tcf7l2
Sbp2l
*
α-Tubulin
B
Tcf7l2
mRNA expression (FPKM)
NC#1
NC#2
Sbp2l
C
D
siNC
siSbp2l
CHX(h)
0
3
6
(kD)
Sbp2l
Tcf7l2
α-Tubulin
E
Relative Tcf7l2 Protein level
1.20
1.00
0.80
0.60
0.40
0.20
0.00
0h
3h
6h
F
Relative expression
Mobp
Plp1
Ccnd1
Jun
P=0.0052
P=5.8E-05
P=0.0016
P=0.041
P=0.018
P=0.0099
P=0.035
siNC
siSbp2l
siSbp2l +Tcf7l2

G

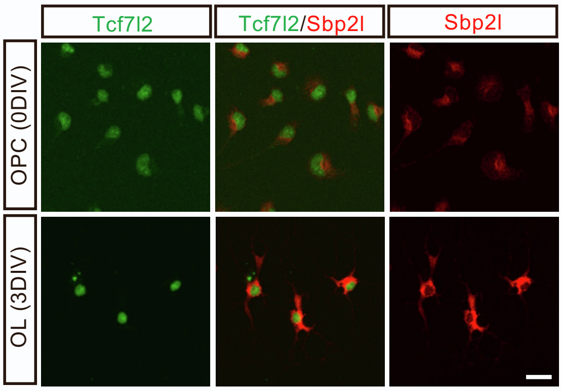

H

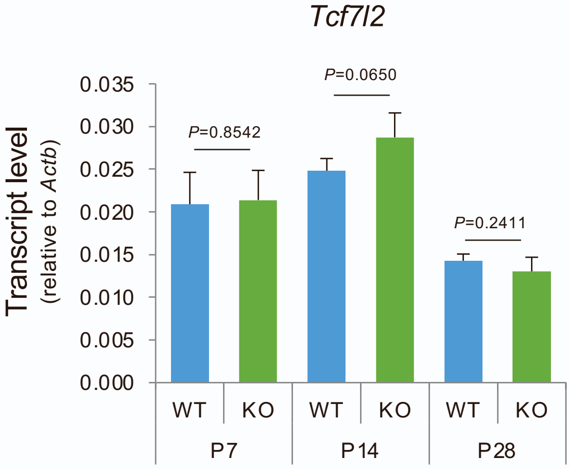

Figure S4. Sbp2l regulates Tcf7l2 at the protein level, related to Figure 5.

A, Western blots confirmed the reduction of Tcf7l2 and Sbp2l protein level in OPC transfected with *siSbp2l.* Related to Figure 5A.

B, RNA-seq analysis revealed that mRNA expression levels (FPKM) of the *Tcf7l2* transcript were not changed in *Sbp2l* KD OPCs. Bar graphs represents means. Each dot represents independent experiment.

C, Secondary structure prediction of the Tcf7l2 3'UTR using RNA structure: a web-based RNA secondary structure prediction tool.

D, Analysis of protein stability by the cycloheximide (CHX) chase assay. OPC were transfected with each siRNA at 3 DIV and treated with 30 µg/ml CHX, an inhibitor of protein biosynthesis. Western blots of cell lysates at 0, 3, 6 hours after CHX treatment were performed to assess the time course changes of Tcf7l2 protein.

E, Graph showing the protein amount of Tcf7l2 relative to an internal control, α-tubulin. Data represent the mean ± SD of three independent experiments.

F, OPC were transfected with each siRNA at 3 DIV together with an expression of Tcf7l2 1 day after siRNA transfection. qRT-PCR assay was performed to assess the effect of Tcf7l2 for their downstream genes. Data represent the mean ± SD of four independent experiments. Student's *t test*.

G, Immunofluorescence using antibodies against Tcf7l2 (green) and Sbp2l (red) at 0 day (OPC 0DIV, top) or 3 days (OL 3DIV, bottom) after induction of OL differentiation. Note that OPC/OLs are expressing both proteins. Scale bar indicates 20 µm.

H, qRT-PCR indicated no difference in Tcf7l2 mRNA in the spinal cord tissues between Sbp2l KO and WT mice. Data represent the mean ± SD of four independent experiments. Welch's *t test*.

A

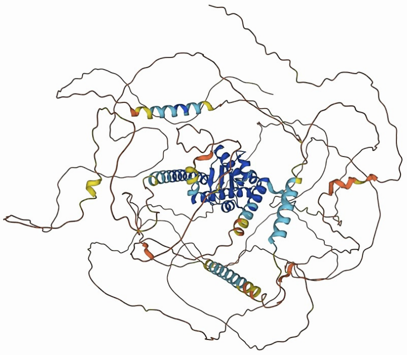

Sbp2l (1089 a.a.)

B

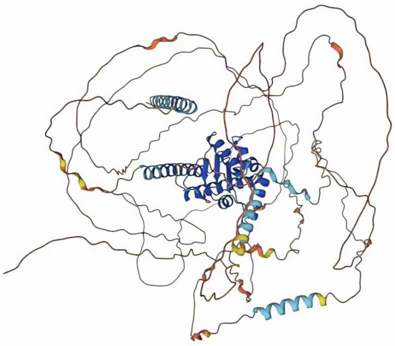

Sbp2 (858 a.a.)

C

Homology of L7Ae domain (69.9% identical a.a)

Score	Expect	Method	Identities	Positives	Gaps
140 bits(353)	4e-50	Compositional matrix adjust.	72/103(70%)	87/103(84%)	0/103(0%)

```
Sbp2l   ERIYQKDPVRAKARRRLVMGLREVTKHMKLNKIKCVIISPNCEKIQSKGGLDEALYNVIA  60
        +R+YQKDPV+AK +RRLV+GLREV KH+KL K+KC+IISPNCEK QSKGGLD+ L+ +I
Sbp2    DRMYQKDPVKAKTKRRLVLGLREVLKHLKLRKLKCIIISPNCEKTQSKGGLDDTLHTIID  60

Sbp2l   MAREQEIPFVFALGRKALGRCVNKLVPVSVVGIFNYFGAESLF  103
         A EQ IPFVFAL RKALGR +NK VPVS+VGIF+Y GA+  F
Sbp2    CACEQNIPFVFALNRKALGRSLNKAVPVSIVGIFSYDGAQDQF  103
```

Figure S5. Protein structure of Sbp2l and Sbp2 protein, related to Figure 5.
A-B, Secondary structure of mouse Sbp2l and Sbp2 protein analyzed by Alpha-Fold. IDs for Sbp2l: AF-Q6A098-F1 and for Sbp2: AF-Q3U1C4-F1 .
C, Amino acid sequence and homology of L7Ae domain of Sbp2l and Sbp2 protein.
